# Reactive oxygen species trigger motoneuron death in non-cell-autonomous models of ALS through activation of c-Abl signaling

**DOI:** 10.3389/fncel.2015.00203

**Published:** 2015-06-09

**Authors:** Fabiola Rojas, David Gonzalez, Nicole Cortes, Estibaliz Ampuero, Diego E. Hernández, Elsa Fritz, Sebastián Abarzua, Alexis Martinez, Alvaro A. Elorza, Alejandra Alvarez, Felipe Court, Brigitte van Zundert

**Affiliations:** ^1^Center for Biomedical Research, Faculty of Biological Sciences and Faculty of Medicine, Universidad Andres BelloSantiago, Chile; ^2^Faculty of Biological Sciences, Pontificia Universidad Católica de ChileSantiago, Chile; ^3^Millennium Institute of Immunology and ImmunotherapySantiago, Chile

**Keywords:** ALS, non-cell-autonomous, motor neuron, mitochondria, reactive oxygen species (ROS), c-Abl

## Abstract

Amyotrophic lateral sclerosis (ALS) is a fatal neurodegenerative disease in which pathogenesis and death of motor neurons are triggered by non-cell-autonomous mechanisms. We showed earlier that exposing primary rat spinal cord cultures to conditioned media derived from primary mouse astrocyte conditioned media (ACM) that express human SOD1^G93A^ (ACM-hSOD1^G93A^) quickly enhances Na_v_ channel-mediated excitability and calcium influx, generates intracellular reactive oxygen species (ROS), and leads to death of motoneurons within days. Here we examined the role of mitochondrial structure and physiology and of the activation of c-Abl, a tyrosine kinase that induces apoptosis. We show that ACM-hSOD1^G93A^, but not ACM-hSOD1^WT^, increases c-Abl activity in motoneurons, interneurons and glial cells, starting at 60 min; the c-Abl inhibitor STI571 (imatinib) prevents this ACM-hSOD1^G93A^-mediated motoneuron death. Interestingly, similar results were obtained with ACM derived from astrocytes expressing SOD1^G86R^ or TDP43^A315T^. We further find that co-application of ACM-SOD1^G93A^ with blockers of Na_v_ channels (spermidine, mexiletine, or riluzole) or anti-oxidants (Trolox, esculetin, or tiron) effectively prevent c-Abl activation and motoneuron death. In addition, ACM-SOD1^G93A^ induces alterations in the morphology of neuronal mitochondria that are related with their membrane depolarization. Finally, we find that blocking the opening of the mitochondrial permeability transition pore with cyclosporine A, or inhibiting mitochondrial calcium uptake with Ru360, reduces ROS production and c-Abl activation. Together, our data point to a sequence of events in which a toxic factor(s) released by ALS-expressing astrocytes rapidly induces hyper-excitability, which in turn increases calcium influx and affects mitochondrial structure and physiology. ROS production, mediated at least in part through mitochondrial alterations, trigger c-Abl signaling and lead to motoneuron death.

## Introduction

Amyotrophic lateral sclerosis (ALS) is a fatal paralytic disorder caused by the progressive degeneration of upper and lower motoneurons during adulthood, and results in death by respiratory failure, usually within 3–5 years of diagnosis. The majority of ALS cases are sporadic (sALS), but ∼10% are familial (fALS) and are generated by mutations in at least 24 identified ALS-associated gene loci, including those for superoxide dismutase (SOD1) and transactive response DNA-binding protein 43 (TDP-43), as well as by hexanucleotide expansion in C9orf72 (Bento-Abreu et al., 2010; DeJesus-Hernandez et al., 2011; Ferraiuolo et al., 2011; Renton et al., 2011, 2014; Wegorzewska and Baloh, 2011; Sreedharan and Brown, 2013). Much of our understanding of ALS, however, is based on a subgroup of fALS patients who carry mutations in the gene that encodes SOD1; the exact mechanisms how SOD1 becomes toxic have not been elucidated (Cleveland and Rothstein, 2001; Pasinelli and Brown, 2006). *In vivo* and *in vitro* studies with transgenic ALS animal models (especially with the SOD1^G93A^ mice) yielded the identification of pathogenic changes in affected motoneurons: these alterations include mitochondrial dysfunction, hyper-excitability, glutamate excitotoxicity, nitroxidative stress from reactive oxygen species (ROS) or reactive nitrogen species (RNS; collectively leading to nitroxidative stress), protein aggregation and misfolding, proteasome impairment, cytoskeletal disruption, activation of cell death signals, and dysregulation of transcription and RNA processing (Cleveland and Rothstein, 2001; Bruijn et al., 2004; Pasinelli and Brown, 2006; Ferraiuolo et al., 2011; Cozzolino et al., 2012; van Zundert et al., 2012).

An increasing number of *in vitro* studies report that astrocytes that express mutant SOD1 selectively kill motoneurons through non-cell-autonomous toxicity (Vargas et al., 2006; Di Giorgio et al., 2007; Nagai et al., 2007; Cassina et al., 2008; Marchetto et al., 2008; Haidet-Phillips et al., 2011; Castillo et al., 2013; Fritz et al., 2013; Meyer et al., 2014; Re et al., 2014; Rojas et al., 2014). Studies of interactions between neurons and astrocytes suggest that similar pathogenic changes occur in human ALS patients and in transgenic ALS models, including those that are based on mitochondrial dysfunction, hyper-excitability, and nitroxidative stress, underscoring the value of these model systems (Cassina et al., 2008; Marchetto et al., 2008; Fritz et al., 2013; Rojas et al., 2014). Despite substantial progress in the identification of pathogenic changes, as well as of the cell types that contribute to them, no cure exists for this profoundly debilitating disease, and the mechanisms that underlie motoneurons death in ALS remain largely unknown; in fact, we do not know even whether the neuronal abnormalities are a primary or secondary event, or whether they result from a compensatory mechanism (van Zundert et al., 2012). In part this is because classical approaches for studying neuron-glial interactions use a co-culture system wherein neurons are grown on a feeder layer of astrocytes, thus masking the temporal interplay between original and secondary pathogenic events.

To circumvent this, we use astrocyte conditioned media (ACM), secreted by primary astrocytes derived from transgenic ALS mouse models (including ACM-SOD1^G93A^), and expose primary wild-type (WT) rat spinal cord cultures (4 DIV) to this ACM for varying times (mins, hours, days). Use of this *in vitro* system, along with electrophysiological recordings, calcium imaging, immunostaining and pharmacology, led us to determine that applying ACM-hSOD1^G93A^, but not ACM-hSOD1^WT^, to primary WT spinal cord cultures rapidly increases the neurons’ evoked action potentials (eAPs; starting at 15–20 min after ACM exposure); this is followed by calcium influx and the generation of intracellular nitroxidative stress (ROS/RNS; starting at 30 min), thereby leading to specific and robust motoneuron death within days (Fritz et al., 2013; Rojas et al., 2014).

Here we wanted to establish the mechanisms whereby ROS/RNS mediates pathogenesis and death of motoneurons, and focused on the interplay between oxidative stress, mitochondrial structure and physiology, and c-Abl activation; these processes are linked to ALS pathology and influenced by ROS. Although the link between oxidative stress and impaired mitochondrial function has been established in diverse ALS model systems (von Lewinski and Keller, 2005; Grosskreutz et al., 2010; Carrì and Cozzolino, 2011; Drechsel et al., 2012; Tan et al., 2014), use of conventional co-culture systems has yielded little about the causal relationship between the stress and the dysfunction. Previous studies also have implicated active c-Abl in a variety of neurodegenerative diseases, including in ALS (Katsumata et al., 2012), Alzheimer’s disease (Alvarez et al., 2004; Cancino et al., 2008; Estrada et al., 2011; Gonzalez-Zuñiga et al., 2014), and Parkinson’s disease (Ko et al., 2010; Imam et al., 2011, 2013).

In addition to its classic function in leukemia pathogenesis, the c-Abl no-receptor tyrosine kinase plays a role in neuronal development and is required for the proper functioning of differentiated neurons (Moresco and Koleske, 2003; Hernández et al., 2004; Bradley and Koleske, 2009). Activated c-Abl has important roles in neuronal cytoskeleton remodeling, promotes dendritogenesis, and regulates adhesion, migration and growth cone path-finding (Koleske et al., 1998; Lu et al., 2002; Rhee et al., 2002; Woodring et al., 2002; Moresco et al., 2003); these processes, as well as cell death, are dependent on the activation c-Abl by of phosphorylation of its tyrosine 245 (Tyr245) and tyrosine 412 (Tyr412; Zhu and Wang, 2004; Gonfloni et al., 2012). In the hippocampus, c-Abl is localized in both the pre- and postsynaptic regions and regulates synaptic structure and function (Moresco et al., 2003; Perez de Arce et al., 2010; Vargas et al., 2014). The contribution of c-Abl signaling activation to neuronal apoptosis has also been reported. For example, it has been shown that c-Abl regulates the choice between cell survival in an arrested state and apoptosis (Wang, 2005), controlling the function and stabilization of p73 in response to genotoxic stress (Tsai and Yuan, 2003). In addition, c-Abl and Cdk5 cooperatively regulate the maximal activation of p53, which results in neuronal apoptosis in response to oxidative stress by hydrogen peroxide (Levav-Cohen et al., 2005; Lee et al., 2008). c-Abl is activated by a wide range of stimuli, including inflammation, DNA damage, amyloid beta, and oxidative stress (van Etten, 1999; Klein et al., 2011; Schlatterer et al., 2011b).

In the present study, we used the ACM-SOD1^G93A^ *in vitro* model system, together with immunostaining, real-time imaging with fluorescent markers for nitroxidative stress and mitochondrial membrane depolarization, electron microscopy, as well as pharmacological treatments, to demonstrate that c-Abl activation and mitochondrial swelling and membrane depolarization play key roles in the pathogenesis and death of motoneurons induced by toxic factor(s) released from SOD1^G93A^-expressing astrocytes. Our findings suggest that mitochondria are an important, but not an exclusive, source of ROS/RNS production which activates the c-Abl signaling pathway. And finally, we use diverse compounds that reduce Na_v_ channel activity and extracellular calcium levels, to unveil that hyper-excitability and calcium influx into the cytoplasm occur upstream of ROS/RNS production, alterations on the mitochondrial structure and membrane potentiation, and c-Abl activation.

## Materials and Methods

### Animals

Care and use of rodents was in accordance with the US National Institute of Health guidelines, and was approved by the Institutional Animal Care and Use Committee of Andres Bello University. Hemizygous transgenic mice carrying mutant human SOD1^G93A^ (high copy number; B6SJL; Cat. No. 002726) and WT human SOD1^WT^ (B6SJL; Cat. No. 002297) were originally obtained from Jackson Laboratories (Bar Harbor, ME, USA). Non-transgenic littermates and transgenic mice over-expressing the gene for human SOD1^WT^ were used as controls. Transgenes were identified by polymerase chain reaction (Wegorzewska et al., 2009; Castillo et al., 2013; Fritz et al., 2013). The SOD1^G93A^ mice, but not the hSOD1^WT^ mice, develop signs of neuromuscular deficits (tremor of the legs and loss of extension reflex of the hind paws) starting at 3 months of age and have an average lifespan of 19–21 weeks (Gurney et al., 1994). Mice carrying SOD1^G86R^ (Ripps et al., 1995) or TDP43^A315T^ (Wegorzewska et al., 2009) develop similar loss of motor function between 3 and 4 months and do not survive to the age of 4 months.

### Conditioned Media Preparation

Astrocyte-onditioned media was prepared as described (Nagai et al., 2007; Castillo et al., 2013; Fritz et al., 2013; Rojas et al., 2014). Briefly, cultures of astrocytes were prepared from P1-2 WT mice and from ALS transgenic mice. Cultures were maintained in DMEM (Hyclone, Cat. No. SH30081.02) containing 10% FBS (Hyclone, Cat. No. SH30071.03; lot ATC31648) and 1% penicillin–streptomycin (Gibco, Cat. No. 15070-063) at 37^∘^C 5% CO_2_. Cultures reached confluence after 2–3 weeks and contained >95% GFAP^+^ astrocytes. Residual microglia were removed by shaking cultures in an orbital shaker (200 r.p.m. in the incubator) overnight (7 h), at which point media was replaced by spinal culture media (see below). After 7 days, ACM was collected, centrifuged (500 *g* for 10 min) and stored at -80^∘^C; before use, it was supplemented with 4.5 mg ml^-1^ D-glucose (final concentration) and penicillin/streptomycin, and filtered. A chick hindlimb muscle extract was also added to the ACM before use (Sepulveda et al., 2010).

While in Nagai et al. (2007) the ACM-hSOD1^G93A^ is added to the cultures undiluted, for all our experiments presented here and previously the ACM-hSOD1^G93A^ as well as the ACM-hSOD1^WT^ was diluted 8–10 fold. The exact dilution was determined for each new batch of ACM by comparing the motoneuron toxicity of the ACM from transgenic animals carrying the ALS-causing mutants (ACM-hSOD1^G93A^, ACM-SOD1^G86R^ or ACM-TDP43^A315T^) to that of ACM generated from mice carrying the WT human SOD1 gene (ACM-hSOD1^WT^) or from non-transgenic littermates; at the selected dilutions the conditioned media derived from the astrocytes expressing the ALS-causing genes robustly killed motoneurons, whereas the control media did not affect motoneuron survival. The ACM was applied to ventral spinal cord cultures derived from rats because better quality motoneurons are obtained from rats than from mice; a number of studies have shown that such mixed species co-cultures (from rat, mice, human) do not appear to induce any side effects (e.g., Pehar et al., 2004; Di Giorgio et al., 2007; Nagai et al., 2007; Castillo et al., 2013; Fritz et al., 2013; Re et al., 2014; Rojas et al., 2014).

### Primary Spinal Cord Neuronal Cultures

Pregnant Sprague-Dawley rats were deeply anesthetized with CO_2_, and primary spinal cultures were prepared from E14 pups (Sepulveda et al., 2010; Fritz et al., 2013; Rojas et al., 2014). Briefly, whole spinal cords were excised and placed into ice-cold HBSS (Gibco, Cat. No. 14185-052) containing 50 μg/ml penicillin/streptomycin (Gibco, Cat. No. 15070-063). The dorsal part of the spinal cord was removed using a small razor blade, and the ventral cord was minced and enzymatically treated by incubating in pre-warmed PBS 1x containing 0.25% trypsin (Gibco, Cat. No. 15090-046) for 20 min at 37^∘^C. Cells were transferred to a 15 ml tube containing neuronal growth media containing 70% MEM (Gibco, Cat. No. 11090-073), 25% Neurobasal media (Gibco, Cat. No. 21103-049), 1% N2 supplement (Gibco, Cat. No. 17502-048), 1% L-glutamine (Gibco, Cat. No. 25030-081), 1% penicillin–streptomycin (Gibco, Cat. No. 15070-063), 2% horse serum (Hyclone, Cat. No. SH30074.03; lot AQH24495) and 100 mM sodium pyruvate (Gibco, Cat. No. 11360-070); they were precipitated, transferred to a new 15-ml-tube containing 2 ml of growth media, re-suspended by mechanical agitation through fire-polished glass Pasteur pipettes of different tip diameters, and counted; 4,8 × 10^6^ cells were plated on freshly prepared poly-L-lysine-coated 24-well plates (1 mg/ml; 30.000–70.000 MW; Sigma, Cat. No. P2636). Cells were cultured for 7 days at 37^∘^C under 5% CO_2_, and supplemented with 45 μg/ml chick leg extract (Sepulveda et al., 2010); the media was refreshed every 3 days.

### Cell Survival Analysis

To measure survival of motoneurons and interneurons, cultures were immunolabeled and counted as previously described (Sepulveda et al., 2010; Castillo et al., 2013; Fritz et al., 2013; Rojas et al., 2014). Briefly, primary spinal cultures were fixed at 7 DIV with 4% paraformaldehyde, and immunostained with a rabbit polyclonal antibody against MAP2 (1:400; Cat. No. sc-20172; Santa Cruz Biotechnology) to label all neurons (interneurons plus motoneurons) and with a mouse monoclonal SMI-32 antibody (1:600, Cat. No. SMI-32R; Sternberger Monoclonals) to reveal the presence of unphosphorylated neurofilament-H, which is expressed specifically in motoneurons in spinal cord cultures (Urushitani et al., 2006; Nagai et al., 2007); previously we found that our WT primary spinal cultures typically contain at least 8–10% motoneurons until 12 DIV (Sepulveda et al., 2010). Fluorescent neurons were visualized with epifluorescent illumination on an Olympus IX81 microscope or on a Nikon C1 confocal microscope on which stacks of 0.50-μm optical sections were acquired through entire neurons. Labeling patterns were documented with a 20x objective and a Q-Imaging Micropublisher 3.3 Real-Time Viewing camera; MAP2- and SMI-32-positive neurons were counted off-line within 20 randomly chosen fields, and the percentage of SMI-32-positive motoneurons within the total number of MAP2-positive cells was calculated. Each condition was replicated in at least three independent cultures and in duplicate.

### Pharmacological Treatments in Culture

Mexiletine (Tocris, Cat. No. 2596) was dissolved in water to 100 mM and used at final concentration of 25 nM. Riluzole (Sigma, Cat. No. R116) was dissolved in distilled water (plus 10% Tween20) at 100 μM, and added to cultures to final concentration of 100 nM. Spermidine (Sigma, Cat. No. S2626) was dissolved in water at 100 mg/ml and added to cultures to a final concentration of 10 μM. Trolox (Sigma, Cat. No. 238813) was dissolved in distilled water at 100 mM and added to cultures to final concentration of 1 μM. Esculetin (Sigma, Cat. No. 17795) was dissolved in dimethyl sulfoxide (DMSO), and added to cultures to final concentration 25 μM. Tiron (Sigma, Cat. No. D7389) was dissolved in distilled water at 100 mM and added to cultures to final concentration of 25 μM. Ru360 (Calbiochem Cat. No 557440) was dissolved in water at 0.5 mg/mL and added to cultures to final concentration 5 μM. Cyclosporin A (CsA), (Sigma Cat. No. 30024) was dissolved in DMSO at 50 mM, and added to cultures to final concentration 10 μM. EGTA (Sigma Cat. No. E3839) was dissolved at 100 mM in NaOH 1M, and added to cultures to a final concentration of 200 μM. All stock solutions were stored at -20^∘^C.

### c-ABL Immunofluorescence Labeling

For identification c-Abl phosphorylation in specific cell types, primary spinal cultures were fixed at 7 DIV with 4% paraformaldehyde, and immunostained with a rabbit polyclonal antibody against MAP2 and SMI-32 antibody, as indicated above in the section “cell survival analysis.” A mouse monoclonal antibody anti-glial fibrillary acidic protein (GFAP; 1:600, Sigma, Cat. No. G393) was also used. For detecting active c-Abl, a mouse monoclonal antibody recognizing phosphorylated Tyr-412 (1:1000 for slices; 1:600 for cultures *in vitro*; 1:2000 for western blot; Sigma; Cat. No. C5240) was used. For immunostainings in tissue, hSOD1^G93A^ (>P120) and hSOD1^G86R^ (P95) transgenic mice were sacrificed, perfused with 4% PFA and sectioned in crytostat obtaining 40 μm slices. All antibody bindings were visualized with the appropriate Alexa fluorescent secondary antibodies (1:500; Life Technologies). Our WT primary spinal cord cultures typically contain at least 6–10% motoneurons until 12 DIV (Sepulveda et al., 2010). Immunolabeled neurons were documented on an upright Olympus Fluoview 1000 confocal microscope (60x oil objective) or a Nikon Eclipse Ti-U microscope equipped with a SPOT Pursuit^TM^ USB Camera CCD (14-bit), Epi-fl Illuminator, mercury lamp and Sutter Smart-Shutter with a lambda SC controller. For quantification of cell number, cultures were photographed using a 20x objective; MAP2- and SMI-32-positive neurons were counted off–line 20 randomly chosen fields, and the percentage of SMI-32-positive motoneurons within the total number of MAP2-positive cells was calculated. Each condition was replicated in at least three independent cultures, and in duplicate. For phospho-c-Abl quantification in cultures, fluorescence intensity was quantified using ImageJ software (NIH, Bethesda, MD, USA). Briefly, cell body was marked manually to set a region of interest. The mean fluorescence was quantified for each cell and the background was subtracted choosing a region without cells. The fluorescence corresponding to control cells were normalized at 1.

### Mitochondrial Membrane Potential Measurements with TMRM

Changes in mitochondrial membrane potential (mtΔΨ) were determined with the potentiometric dye tetramethyl rhodamine methyl ester, TMRM (Molecular Probes, Cat. No. T-668). At 4 DIV primary ventral spinal cord cultures were washed twice with Hanks solution (Invitrogen, Cat. No. 4025134), loaded in an incubator for 30 min at 37^∘^C, 5% CO_2_ and in the dark with 50 nM of the TMRM dye diluted in Hanks solution. Immediately after, the fluorescent signal was acquired at two time points: before (time 0 min) and 30 min following ACM-hSOD1^G93A^ addition. The excitation and emission wavelengths of TMRM fluorescent dye are 550 and 575 nm, respectively. The pictures were taken using Olympus IX81 (Olympus) microscope equipped with a digital camera Orca-R2 (Hamatsu) at the 100X magnification. Scale bar 20 μm. At least three independent fields were acquired for each condition and at least 10 cells were used for quantification of the fluorescence signal. Cells were marked by drawing a region of interest around the cell body, and mean fluorescence intensity was calculated for each cell after subtraction of the background signal using the image analysis module in ImageJ software.

### Nitroxidative Stress Measurements with CM-H_2_DCF-DA

The intracellular levels of ROS/RNS were measured with CM-H_2_DCFDA (Invitrogen, Cat. No. C6827) as previously described (Rojas et al., 2014). H_2_DCF-DA is not a specific probe for a specific oxidant and has been used to monitor certain ROS/RNS (see Discussion). The CM-H_2_DCF-DA stock solution (1 mM) was prepared in DMSO and was diluted in the culture medium to a final concentration of 1 μM just before addition to the cells. After application of the diverse ACMs to the spinal cord cultures for different time (minutes-hours-days), cells were washed (PBS 1x) to remove the ACMs and exposed to CM-H_2_DCF-DA for 30 min at 37^∘^C in dark, to label both motoneurons and interneurons. To facilitate the CM-H_2_DCF-DA membrane penetration, 0.004% Pluronic acid F-127 (Invitrogen, Cat. No. P-3000MP) was added to the culture medium to facilitate dye entry, eliminate possible hydrolysis of dyes by external esterases and maintain better cell integrity (Appaix et al., 2012). After the incubation time, the CM-H_2_DCF-DA-cointaing culture medium was removed and cultures were washed twice with PBS 1X and suspended in culture medium (500 μl final volume). Next, cells were immediately imaged using an upright Nikon Eclipse Ti-U microscope equipped with a SPOT Pursuit^TM^ USB Camera CCD (14-bit), Epi-fl Illuminator, mercury lamp and Sutter Smart-Shutter with a lambda SC controller. Cells were photographed using a 20x objective. As CM-H_2_DCF-DA is a non-fluorescent dye it passively diffuses into cells and is hydrolyzed intracellularly to the DCFH carboxylate anion that is trapped inside; oxidation of DCFH results in the formation of the fluorescent product DCF, with excitation and emission wavelengths λ_ex_/λ_em_ = 492–495/517–527 nm. The exposure time was kept below 4 s in order to avoid photo-oxidation of the ROS/RNS sensitive dye and for all given treatments fields were exposed for exactly the same amount of time. At least three independent fields were acquired for each condition and at least 10 cells per field were used for quantification of the fluorescence signal. Cells were masked by drawing a region of interest around the cell body, and mean fluorescence intensity was calculated for each cell after subtraction of the background signal using the image analysis module in ImageJ software. All cells, independent of ttheir relative intensity unit (RIU), were included in the analysis. Cultures were also incubated with H_2_O_2_ (200 μM for 20 min) to serve as a positive control.

### Electron Microscopy

For electron microscopy analysis, ventral spinal cord cultures were processed as previously described (Villegas et al., 2014). Briefly, cells were fixed at 4^∘^C o.n. with ultrastructure solution (2.5% glutaraldehide; 0.1 M picric acid; 0.05 M cacodylate buffer; pH 7.4). Cultures were wash in the same buffer, immersed in 1% OsO4 for 1 h followed by a 2 h in block incubation with 2% uranyl acetate. Cultures were then dehydrated using a graded series of ethanol, propylene oxide and infiltrated with Epon (Ted Pella Inc.). Ultrathin sections were contrasted with 1% uranyl acetate and lead citrate. Grids were observed with a Philips Tecnai 12 electron microscope operated at 80 kV. Negative films were developed and scanned.

### Western Blot

The spinal cord and brain from post-symptomatic hSOD1^G93A^ and control WT mice were lysed in RIPA buffer (50 mM Tris-HCL pH 7,4; 1% NP-40; 0,5% Na-deoxycholate, 0,1% SDS, 150 mM NaCl and 2 mM EDTA) with protease inhibitor cocktail (Roche, Complete min tables Cat. No. 11836153001) and phosphatase inhibitors [10 mM sodium orthovanadate and 50 mM sodium fluoride (New England Biolabs, Ipswich, MA, USA)]. Protein concentrations were determined using micro BCA Protein Assay Kit (Thermo Scientific, Rockford, IL, USA). Protein samples (30 μg per lane loaded) were separated by 10% SDS-polyacrilamide gel electrophoresis (PAGE) and then transferred to a nitrocellulose membrane (Thermo Scientific, Rockford, IL, USA). Primary antibodies were used at following dilutions: unphosphorylated anti-c-Abl, 1:1000 (Santa Cruz, USA Cat. No. sc-56887); anti-n-cadherin 1:1000 (Santa Cruz, USA, Cat. No. sc-271386). For detecting active c-Abl, rabbit polyclonal antibody recognizing phosphorylated Tyr-412 (1:2000; Sigma; Cat No C5240) were used. Secondary antibodies were used at 1:2000 and detection was performed using ECL Western Blotting Substrate (Thermo Scientific, Rockford, IL, USA). Anti-phospho-c-Abl was diluted in 5% fat-free milk in TBS plus 0.5% Tween-20 (Winkler, Chile), otherwise 5% fat-free milk in PBS plus 0.5% Tween-20 was used.

### Data Analysis

ANOVA, followed by *post hoc* Tukey tests, was used to detect significant changes. Student’s *t*-tests were used to compare the response of two cell populations to individual treatments. Unless otherwise stated, error bars represent the mean ± SEM; ^∗^*p* ≤ 0.05, ^∗∗^*p* ≤ 0.01, ^∗∗∗^*p* ≤ 0.001 vs. control; #*p* < 0.05, ##*p* < 0.01, ###*p* < 0.001 compared to ACMs.

## Results

### ACM-hSOD1^G93A^ Rapidly Increases Phosphorylation of c-Abl in Motoneurons, Interneurons and Astrocytes

We first examined whether c-Abl is phosphorylated in the central nervous system of symptomatic mice that express hSOD1^G93A^. Immunostaining assays as well as western blots of whole lysate extracts from the spinal cord and brain were used to determine the expression of native c-Abl proteins and of c-Abl that is phosphorylated on tyrosine 412 (Tyr412; a site that enhances c-Abl catalytic activity; Hantschel and Superti-Furga, 2004). In agreement with a previous study (Katsumata et al., 2012), we found a robust increase in the phosphorylation of c-Abl in the brain and spinal cord of symptomatic hSOD1^G93A^ mice (P120), compared to non-transgenic littermates; however, unlike this earlier study, our samples did not show an increase in the levels of expression of native c-Abl (Supplementary Figure S1). Interestingly, we also found similar increases in c-Abl phosphorylation levels in the spinal cord and motor cortex of symptomatic SOD1^G86R^ mice (P95; Supplementary Figure S2).

**FIGURE 1:**
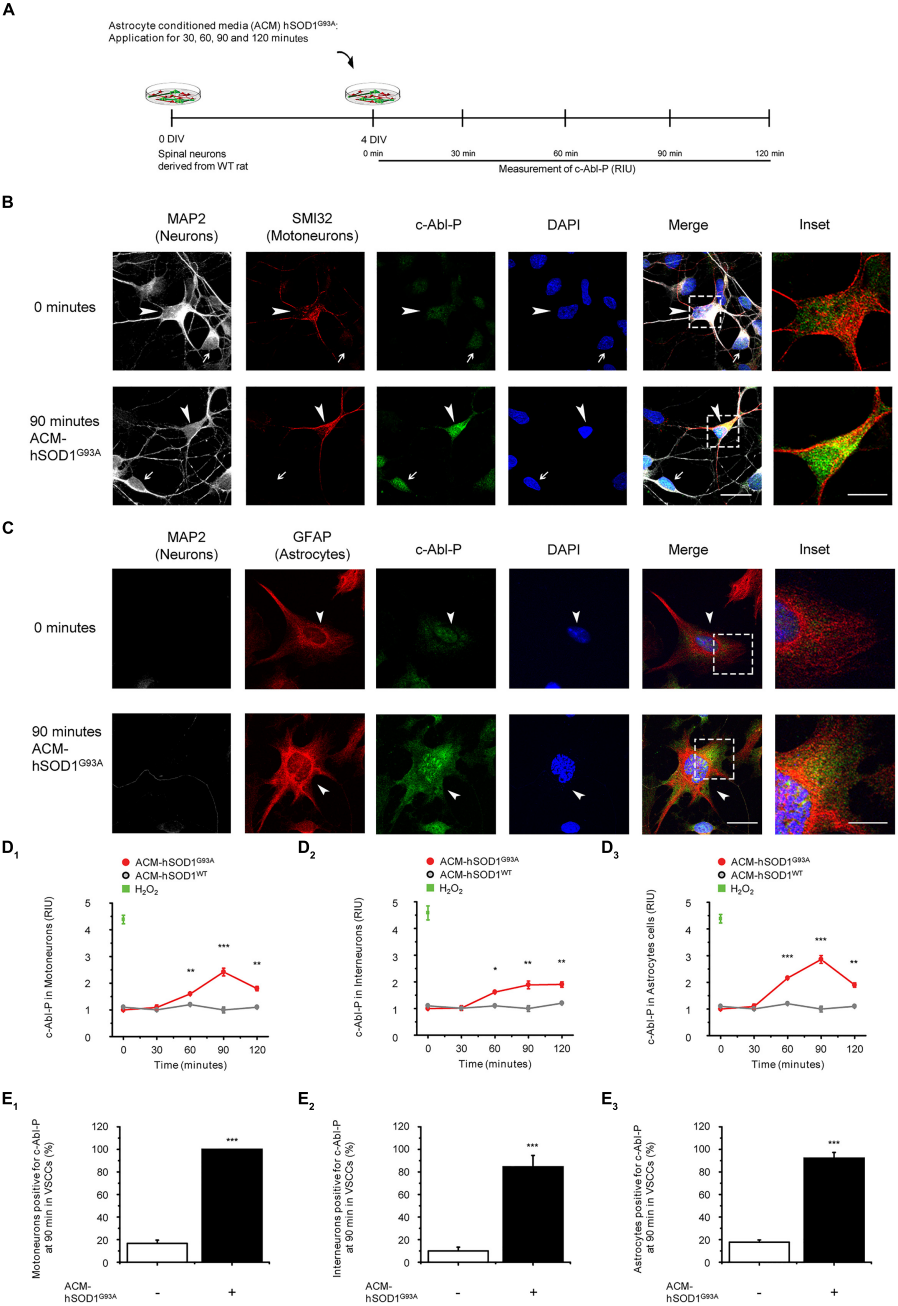
**Exposure of primary spinal cord cultures to ACM-SOD1^**G93A**^ induces increases in c-Abl phosphorylation. (A)** Flow diagram of experiment. Primary wild-type (WT) rat spinal cord cultures (4 DIV) were exposed to ACM derived from transgenic mice overexpressing SOD1^G93A^ (ACM-SOD1^G93A^) for 0–120 min. Next cultures were washed, fixed and immunostained with an antibody recognizing phosphorylated c-Abl (Tyr-412) and cell markers to define cell type. **(B)** Spinal cord cultures untreated (0 min; upper images) or treated with ACM-SOD1^G93A^ for 90 min (lower images) were triple-labeled with anti-microtubule-associated protein 2 (MAP2) antibody (white) to visualize neurons (arrow show interneuron that is MAP2^+^/SMI32^-^), with the SMI-32 antibody (red) to identify motoneurons (arrowhead), and with the phospho-c-Abl antibody (green) to show active c-Abl. Cultures were also stained with DAPI to visualize their nucleus. Inset shows selected motoneuron in the merge. Scale bar, 25 μm. **(C)** Spinal cord cultures untreated (0 min; upper images) or treated for 90 min with ACM-SOD1^G93A^ (lower images) were triple-labeled with anti-MAP2 antibody (white) to visualize neurons (arrows), with the GFAP antibody (red) to identify astrocytes (arrowhead), and with the phospho-c-Abl antibody (green) to show active c-Abl. Inset shows selected astrocyte in the merge. Scale bar, 25 μm. **(D)** Graphs showing the c-Abl-P fluorescent intensity (relative intensity unity; RIU) at 0, 30, 60, 90, and 120 min after application of ACM-SOD1^G93A^ (red line): immunestaining was used to identify c-Abl-P within a particular cell type, including in motoneurons (**D_**1**_**; MAP2^+^/SMI32^+^), interneurons (**D_**2**_**; MAP2^+^/SMI32^-^), and glial cells (**D_**3**_**; MAP2^-^/GFAP^+^). Results obtained ACM-SOD1^WT^ are also included (gray lines). In all experiment, H_2_O_2_ (200 μM for 20 min) served as positive control. Note that c-Abl-P fluorescence peaked in all three cell types after 90 min of incubation with ACM-SOD1^G93A^. **(E)** Graphs showing the percentage of motoneurons **(E_**1**_)**, interneurons **(E_**2**_)**, and glial cells **(E_**3**_)** positive for c-Abl-P after 90 min (at peak) of exposure to ACM-SOD1^G93A^. Values represent mean ± SEM from at least three independent experiments performed in duplicate, analyzed by ANOVA **(D)** or *t*-test **(E)**. ^∗^*p* < 0.05, ^∗∗^*p* < 0.01, ^∗∗∗^*p* < 0.001 vs. control.

To investigate whether soluble toxic factor(s) released by astrocytes that carry the hSOD1^G93A^ mutation can trigger the activation of c-Abl, we exposed 4 DIV ventral spinal cord cultures to ACM-hSOD1^G93A^, fixed the cells at different post treatment times (0–120 min), and immunostained with an antibody against phosphorylated c-Abl (Tyr412; **Figure 1A**). To assess which cell type in our model system displays phosphorylated c-Abl, we used an antibody against microtubule-associated protein 2 (MAP2; recognizing interneurons and motoneurons), the SMI-32 antibody (recognizing motoneurons in ventral spinal cord cultures), and an antibody against the glial fibrillary acidic protein (GFAP; expressed in astrocytes). Analysis of the immunostaining revealed that in ventral spinal cord cultures at 4 DIV, ∼4% of the cells are motoneurons (MAP2^+^/SMI32^+^/GFAP^-^), ∼37% are interneurons (MAP2^+^/SMI32^-^/GFAP^-^), and ∼59% are astrocytes (MAP2^+^/SMI32^-^/GFAP^-^; data not shown). Using this staining strategy to identify cell types, we show that acute application of ACM-hSOD1^G93A^ to 4 DIV spinal cord cultures induces a robust phosphorylation of c-Abl in motoneurons (arrowheads and inset in **Figure 1B**) and interneurons (arrows in **Figure 1B**) and astrocytes (arrowheads and insets in **Figure 1C**). Quantification of fluorescence shows that intensity speaks for all three cell types at 90 min of ACM-hSOD1^G93A^ exposure (red lines in graphs **Figures 1D**_**1**-**3**_). Strong c-Abl phosphorylation was also produced in spinal cord cultures by short-term application of H_2_O_2_ (200 μM for 20 min; green squares in **Figures 1D**_**1**-**3**_). Additional analysis of the number of cells that were immunopositive for phosphorylated c-Abl revealed that following 90 min of exposure to ACM-hSOD1^G93A^ all motoneurons (100%; **Figure 1E**_**1**_), plus the vast majority of interneurons (>80%; **Figure 1E**_**2**_) and astrocytes (>90%; **Figure 1E**_**3**_) expressed the activated form of c-Abl. Analysis of the subcellular distribution of phosphorylated c-Abl further revealed that ACM-hSOD1^G93A^ leads to a homogenous distribution of this activated tyrosine kinase in all three cell types (**Figures 1C–E**_**1**_; insets). Of note is that increases in activated c-Abl also occurs in the cell’s nucleus where it can perform its pro-apoptotic function and repress neuronal genes expression (Zhu and Wang, 2004; Yoshida, 2008; Gonzalez-Zuñiga et al., 2014). Interestingly, we also detected similar increases in c-Abl phosphorylation levels in motoneurons that were subjected to ACM derived from astrocytes expressing SOD1^G86R^ or TDP43^A315T^ (Supplementary Figure S3). In contrast, control ACM obtained from astrocytes that are harvested from transgenic mice carrying the non-pathological WT human SOD1 gene (ACM-hSOD1^WT^) did not induce c-Abl phosphorylation in any of the three cell types (**Figures 1D**_**1**-**3**_; gray lines).

### Treatment of Cultures with c-Abl Inhibitor STI571 Prevents Motoneuron Cell Death Induced by ACM-hSOD1^G93A^

**FIGURE 2:**
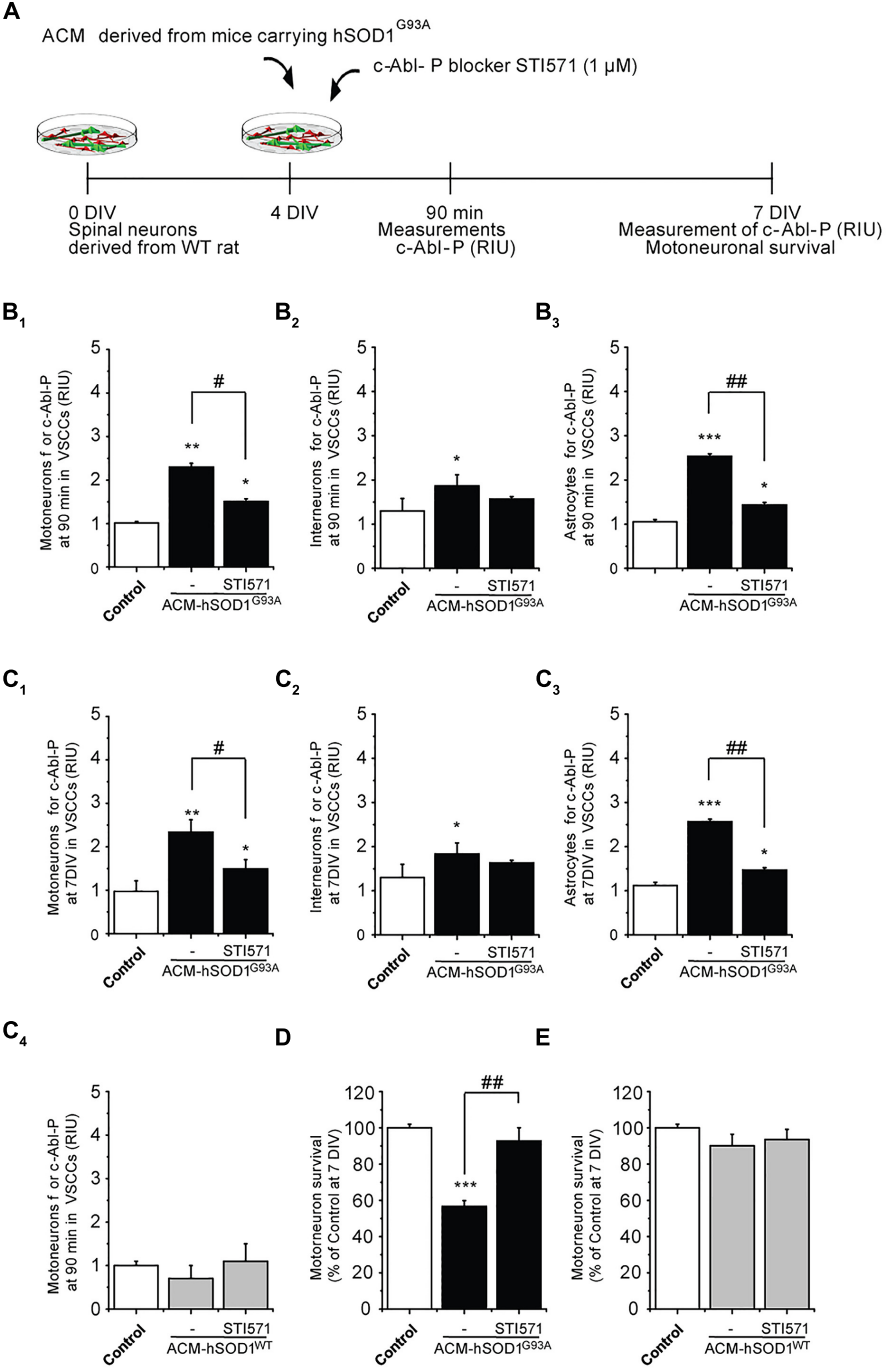
**c-Abl kinase inhibitor STI571 prevents motoneuron death induced by ACM-SOD1^G93A^. (A)** Flow diagram of experiment. ACM-SOD1^G93A^ was applied to 4 DIV spinal cord cultures acutely (for 90 min when c-Abl-P peaks; see **Figure 1**) or chronically (3 days) either alone or in the presence of c-Abl kinase inhibitor STI571 (1 μM). Phosphorylation of c-Abl was measured at 4 and 7 DIV. Cell survival was measured at 7 DIV. **(B)** Graphs showing fluorescence intensities (RIU) for c-Abl-P at 4 DIV when treated acutely (90 min) with ACM-SOD1^G93A^ alone or ACM-SOD1^G93A^ plus STI571; motoneurons **(B_**1**_)**, interneurons **(B_**2**_)** and glial cells **(B_**3**_)** were identified by immunostaining (as in **Figure 1**). **(C_**1**-**3**_)** Same as in **(B)**, but c-Abl-P is measured at 7 DIV when treated chronically (3 days) with ACM-SOD1^G93A^ alone or with STI571. **(C_**4**_)** c-Abl-P is measured at 7 DIV when treated chronically (3 days) with ACM-SOD1^WT^ alone or with STI571. **(D,E)** Graphs showing the relative percentage of motoneurons that survived at 7 DIV, after being treated with STI571 and ACM-SOD1^G93A^ **(D)** or ACM-SOD1^WT^ **(E)**. Values represent mean ± SEM from at least three independent experiments performed in duplicate, analyzed by One-Way ANOVA followed by a Tukey *post hoc* test. ^∗^*p* < 0.05, ^∗∗^*p* < 0.01, ^∗∗∗^*p* < 0.001 relative to control conditions. #*p* < 0.05, ##*p* < 0.01 compared to survival with the ALS-causing ACM to at 7 DIV without STI571.

To determine whether the increased phosphorylation of c-Abl induced by ACM-hSOD1^G93A^ contributes to motoneuron death, 4 DIV spinal cord cultures were co-incubated with ACM-hSOD1^G93A^ plus the c-Abl kinase inhibitor STI571, and motoneuron survival (MAP2^+^/SMI32^+^) was assessed at 7 DIV (**Figure 2A**). We first used immunostaining (as in **Figure 1**) to assess the effectiveness of STI571 in preventing c-Abl phosphorylation in the three spinal cord cell types. STI571, also called imatinib, when applied at micromolar levels to primary neuronal cultures effectively inhibits the phosphorylation of c-Abl following various stimuli, including oxidative stress (Alvarez et al., 2004, 2008; Cancino et al., 2011; Klein et al., 2011). We found that 4 DIV cultures treated for 90 min with both ACM-hSOD1^G93A^ and 2 μM STI571 displayed a significant reduction in the intensity of immunoreactivity for phosphorylated c-Abl (**Figures 2B**_**1**-**3**_) and in the percentage of cells that were positively stained (Supplementary Figures S4B_1-3_); these changes were observed for motoneurons (**Figure 2B**_**1**_), interneurons (**Figure 2B**_**2**_), and astrocytes (**Figure 2B**_**3**_). Following chronic treatment of the cultures with ACM-hSOD1^G93A^ plus STI571, and analyzed 3 days later (at 7 DIV), we found that the reduction in intensity (**Figures 2C**_**1**-****3****_) and in the percentage of cells positive for phosphorylated c-Abl (Supplementary Figures S4C_**1**-**3**_) was maintained in all three cell types. Importantly, application of STI571 was able to prevent motoneuron cell death induced by SOD1^G93A^ (**Figure 2D**). Similarly, STI571 prevented also motoneuron cell death induced by SOD1^G86R^ or TDP43^A315T^ (Supplementary Figures S3C–E). These results document that activation of c-Abl in two completely unrelated ALS models leads to the death of motoneurons. In contrast to the ACMs obtained from ALS astrocytes, chronic application of STI571 with control ACM-hSOD1^WT^ did not alter c-Abl intensity in motoneurons (**Figure 2C**_**4**_) or number of surviving motoneurons (**Figure 2E**). Collectively, these results indicate that the favorable effects of STI571 are not a result of a non-specific beneficial influence of this compound, but rather that the inhibitor drug counterbalances the toxic effects of ACM-hSOD1^G93A^. Because ACM-hSOD1^WT^ did not induce a significant change in the phosphorylation of c-Abl, in motoneuron cell survival, or in ROS/RNS production (as also shown previously; Fritz et al., 2013; Rojas et al., 2014), results from use of the control ACM are omitted from most figures.

### Nitroxidative Stress Induced by ACM-hSOD1^G93A^ Leads to Activation of c-Abl

**FIGURE 3:**
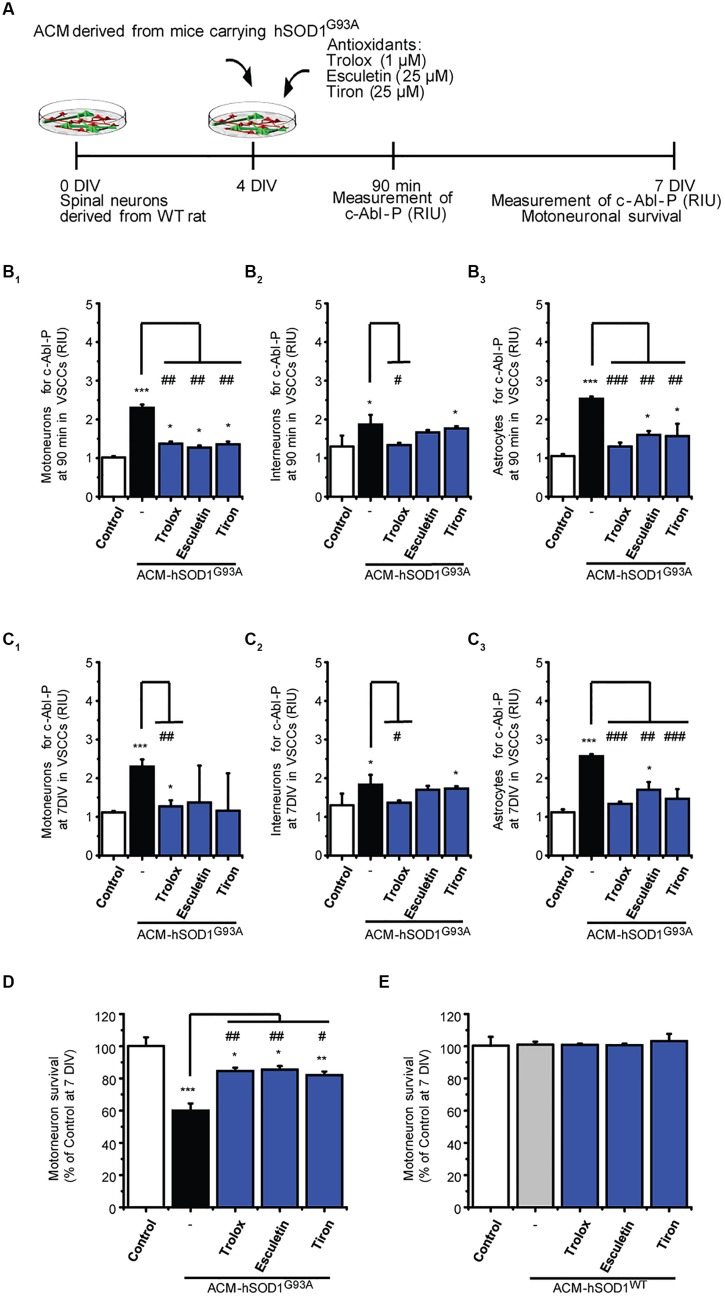
**Antioxidants reduce phosphorylation of c-Abl and prevent motoneuron death induced by ACM-SOD1^G93A^. (A)** Flow diagram of experiment. ACM-SOD1^G93A^ was applied to 4 DIV spinal cord cultures acutely (for 90 min when c-Abl-P peaks; see **Figure 1**) or chronically (3 days), either alone or in the presence of antioxidants Trolox (1 μM), esculetin. (25 μM), or tiron (25 μM) Phosphorylation of c-Abl was measured at 4 and 7 DIV. Cell survival was measured at 7 DIV. **(B)** Graphs showing fluorescence intensities (RIU) for c-Abl-P at 4 DIV when treated acutely (90 min) with ACM-SOD1^G93A^ alone or ACM-SOD1^G93A^ plus the antioxidants; motoneurons **(B_**1**_)**, interneurons **(B_**2**_)** and glial cells **(B_**3**_)** were identified by immunostaining (as in **Figure 1**). **(C)** Same as in **(B)**, but c-Abl-P is measured at 7 DIV when treated chronically (3 days) with ACM-SOD1^G93A^ alone or in the presence of the antioxidants. **(D,E)** Graphs showing the relative percentage of motoneurons that survived at 7 DIV, after being treated with antioxidants and ACM-SOD1^G93A^ **(D)** or ACM-SOD1^WT^ **(E)**. Values represent mean ± SEM from at least 3 independent experiments performed in duplicate, analyzed by One-Way ANOVA followed by a Tukey *post hoc* test. ^∗^*p* < 0.05, ^∗∗^*p* < 0.01, ^∗∗∗^*p* < 0.001 relative to control conditions. #*p* < 0.05, ##*p* < 0.01, ###*p* < 0.001 compared to survival with the ALS-causing ACM to at 7 DIV without antioxidants.

Next we investigated possible mechanisms underlying the induction of c-Abl activation by ACM-hSOD1^G93A^. Because c-Abl is activated by oxidative stress and induces cell death (van Etten, 1999; Klein et al., 2011; Schlatterer et al., 2011b), we focused on the role of ROS/RNS. Indeed, we find that H_2_O_2_–induced oxidative stress caused a strong phosphorylation of c-Abl in motoneurons, interneurons and astrocytes (see green squares in **Figure 1D**_**1**-**3**_). Conversely, we wanted to determine whether co-application of antioxidants with the toxic ACM-hSOD1^G93A^ can prevent c-Abl activation and motoneuron cell death. Keeping in mind that antioxidants can be used as a therapeutic strategy for ALS, we tested several different types of compounds with antioxidant and/or free scavenger capacities, that also have the biochemical properties to effectively penetrate the blood–brain-barrier: these include Trolox (a H_2_O soluble vitamin E analog), esculetin, and 4,5-dihydroxy-1,3-benzenedisulfonic (tiron). Trolox and esculetin were selected because they were identified in a large screening assay as having strong antioxidant activity, and as protecting the viability of mutant SOD1^G93A^-expressing cell lines under stress conditions (Barber and Shaw, 2010). In a previous study, we tested multiple doses (ranging from 100 to 100 μM), and found that 1 μM Trolox and 25 μM esculetin reduced intracellular levels of ROS/RNS, and largely prevented primary motoneuron cell death induced by ACM-hSOD1^G93A^ (**Figure 3D**), without affecting the survival of control motoneurons (Rojas et al., 2014; and shown in **Figure 3E**). We also tested tiron, because it permeabilizes the mitochondrial membrane and is more effective in reducing oxidative stress compared to the mitochondria-targeted antioxidant MitoQ (Oyewole et al., 2014). We assessed the antioxidant capacity and toxicity of multiple doses (ranging from 1 μM to 1 mM) of this antioxidant (not shown), and found that 25 μM tiron strongly reduces H_2_O_2_–induced oxidative stress (Supplementary Figure S5) in the absence of cell death of control motoneurons (**Figure 3E**).

Notably, all three antioxidants effectively abrogated the phosphorylation of c-Abl in motoneurons, interneurons and astrocytes that is induced by acute (90 min exposure and tested at 4 DIV; **Figures 3B**_**1**-**3**_) or chronic (3 days exposure and tested at 7 DIV; **Figures 3C**_**1**-**3**_) treatment with ACM-hSOD1^G93A^ (**Figures 3B,C** show c-Abl-P intensities, while the percentage of cells positive for c-Abl-P is shown in Supplementary Figure S6). Importantly, motoneuron death was also largely prevented following chronic co-application of ACM-hSOD1^G93A^ and Trolox, esculetin, or tiron (**Figure 3D**), whereas no increase in the number of motoneurons was observed under control conditions with use of any of these antioxidants (**Figure 3E**). Together, our data indicate that counterbalancing the production of ACM-hSOD1^G93A^-induced ROS/RNS protects motoneurons from death by diminishing, at least in part, c-Abl activation.

### ACM-hSOD1^G93A^ Leads to Mitochondrial Swelling and Membrane Depolarization

**FIGURE 4:**
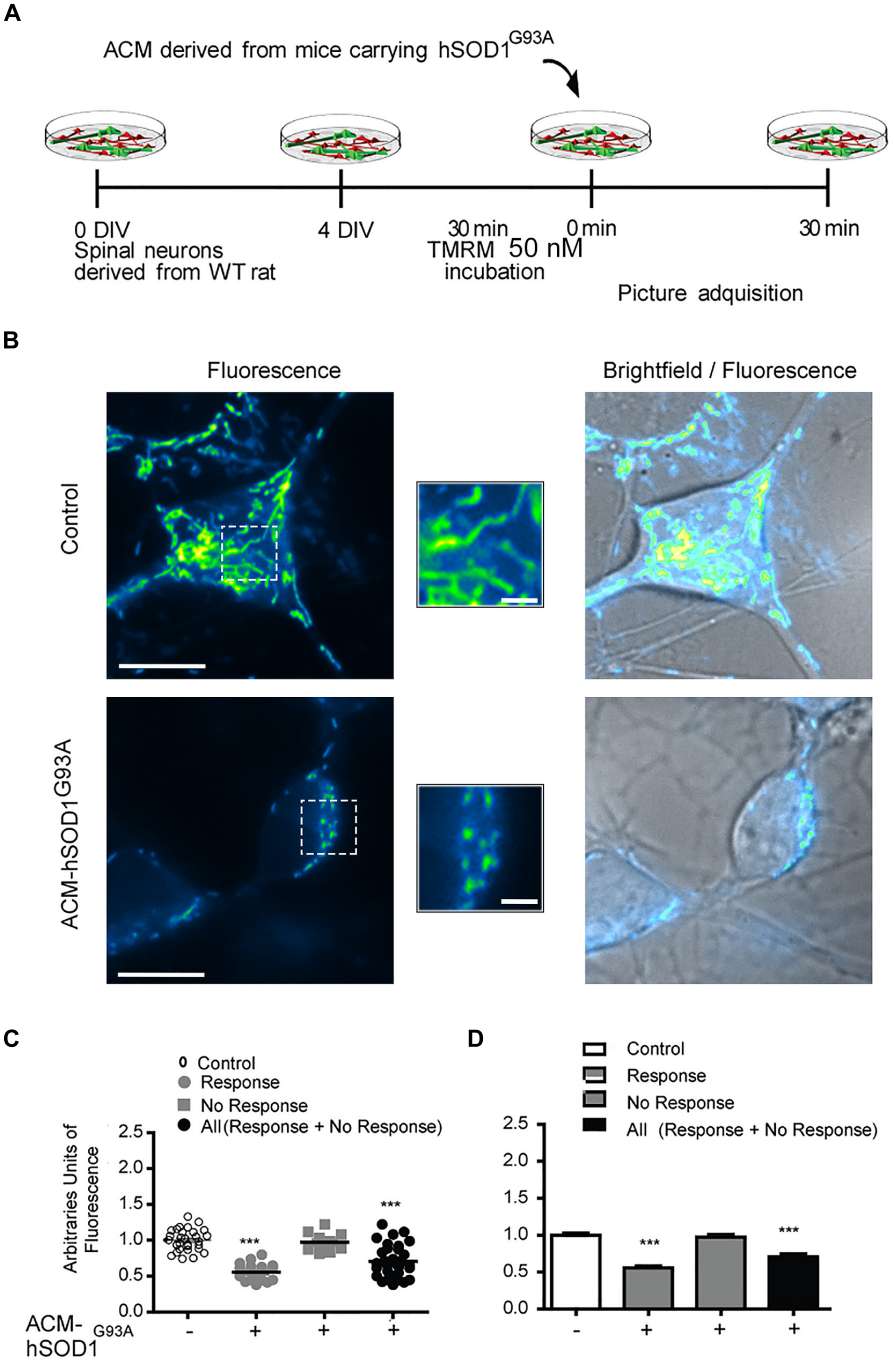
**Mitochondrial potential membrane change after ACM-hSOD1^G93A^. (A)** Flow diagram of experiment. Four DIV spinal cord cultures were incubated with TMRM (50 nM) during 30 min and then exposed to ACM-hSOD1^G93A^ for 30 min. Pictures were acquired using a combination of real-time fluorescence and phase-contrast imaging. **(B)** Representative pictures of an untreated neuron (0 min; upper images) or treated for 30 min with ACM-SOD1^G93A^ (lower images). Fluorescence images, brightfield images and their merges are also shown. Scale bar in main pictures 10 and 2 μm in insets. **(C)** Scatter plot showing the percentage of TMRM fluorescence (arbitrary units) in every individual neuron measured under the conditions indicated. Note that the majority (63%) of the ACM-hSOD1^G93A^-treated neurons show a decrease in TMRM fluorescence relative to the control condition, but that another neuronal population (37%) does not. For clarity, we show all neurons (response + no response), those that show reduced TMRM fluorescence after ACM-hSOD1^G93A^ exposure (response) and those that did not displayed TMRM alterations (no response). **(D)** Graph showing the same data as in C. Values represent mean ± SEM from at least three independent experiments performed in duplicate, analyzed by One-Way ANOVA followed by a Tukey *post hoc* test. ^∗∗∗^*p* < 0.001 relative to control conditions.

**FIGURE 5:**
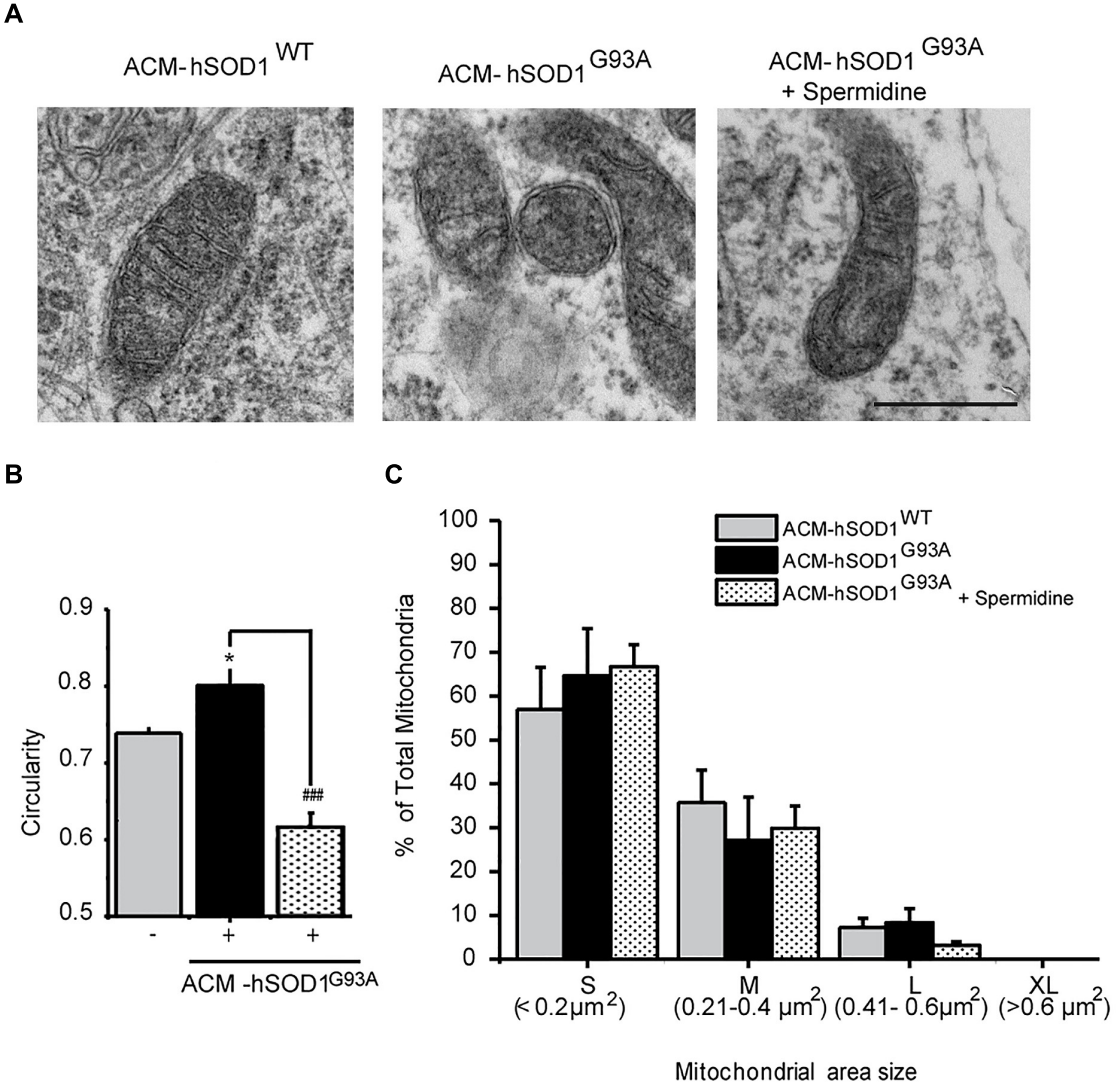
**Exposure of primary spinal cord cultures to ACM-hSOD^G93A^ induces mitochondria morphological alterations. (A)** Representative electron microscopy images of spinal cord cultures treated for 4 h with ACM-hSOD1^WT^, ACM-hSOD1^G93A^ alone or ACM-hSOD1^G93A^ plus spermidine (1 μM). Scale bar, 0.1 μm. **(B)** Graph showing mitochondrial circularity of cells after treatment with the different ACMs with or without spermidine. **(C)** Graph showing percentage of total mitochondria size area of cultures under the same treatment shown in **B**, classified in four groups: “small” (S; < 0.2 μm^2^), “medium” (M; 0.21–0.4 μm^2^), “large” (L; 0.41–0.6 μm^2^) and “extra large” (XL; >0.6 μm^2^). Values represent mean ± S.E.M. from over 100 mitochondria per condition, analyzed by one-way ANOVA followed by a Tukey *post hoc* test. ^∗^*p* < 0.05 relative to ACM-hSOD1^WT^. ###*p* < 0.01 relative to ACM-hSOD^G93A^ without spermidine.

Because dysfunctional mitochondria constitute a major source for elevating ROS production, and because mitochondrial damage is a common feature in ALS patients and in mouse models of ALS (Manfredi and Xu, 2005; Jaiswal et al., 2009; Grosskreutz et al., 2010; Tan et al., 2014), we wanted to know whether toxic factors released by hSOD1^G93A^-expressing astrocytes induce mitochondrial defects in the neurons of our culture system. We first analyzed the morphology and membrane potential of mitochondria in 4–5 DIV spinal cord neurons under control conditions. To fluorescently label mitochondria, we used Tetramethylrhodamine Methyl Ester, Perchlorate (TMRM; **Figure 4**); live time-lapse fluorescence microcopy imaging was accompanied by phase-contrast images to select motoneurons on the basis of their large soma (>20 μm) and extent at least five primary dendrites. The TMRM fluorescent labeling methods indicate that under basal conditions, mitochondria in interneurons and motoneurons of 4–5 DIV spinal cord cultures are mainly organized into a tubular network (especially in the soma), with a few isolated round mitochondria also visible (**Figure 4B**). To get direct evidence for this, we visualized the ultra-structure of mitochondria using transmission electron microscopy (**Figure 5**); it was revealed that neurons in control 4–5 DIV spinal cord cultures display prominent mitochondria, most with an elongated shape and fewer with a globular morphology (see Materials and Methods for identifying neuronal vs. glial mitochondria).

We also studied the membrane potential and morphology of mitochondria of 4–5 DIV spinal cord neurons that were exposed to ACM-hSOD1^G93A^ for different times (minutes to hours). TMRM staining showed that already short-term exposure (30 min) of spinal cord neurons to ACM-hSOD1^G93A^ induces a decrease in the fluorescent labeling in the majority (but not all) of the neurons (**Figure 4B**). Quantification reveals that ∼60% of the neurons displayed a robust reduction in the levels of TMRM fluorescence (see **Figures 4C,D** for scatter plots and graph with average data, respectively); it is not clear why the TMRM signal in another population of neurons (∼40%) remained unchanged. Because the trapping of the TMRM dye inside the mitochondria matrix depends on the mitochondrial membrane potential (ΔΨm), the loss of staining could be a consequence of mitochondrial membrane depolarization, which can be induced by activation of uncoupling proteins, or by the opening of the mitochondrial permeability transition pore (mPTP); alternatively, it could be the result of mitochondria degeneration. The results of two independent sets of experiments support a depolarization event: (i) use of mitoDsRed2 (a marker of the mitochondrial matrix used to assess mitochondrial morphology and that is insensitive to ΔΨm) resulted in no obvious reduction in the number or intensity of labeled mitochondria in cells treated with ACM-hSOD1^G93A^ for 0–90 min (not shown), indicating that mitochondria were not lost; and (ii) ultra-structural analysis did not reveal any reduction in the number of mitochondria in neurons that had been exposed to ACM-hSOD1^G93A^ for 4 h (**Figure 5**). Electron microscopy images, however, did show that exposure to ACM-hSOD1^G93A^ induces mitochondrial alterations within neurons with many mitochondria displaying a subtle, but significant, more swollen morphology (**Figures 5A,B**); this kind of morphology is usually associated with the opening of the mPTP (Ruiz et al., 2014). These observations collectively indicate that ACM-hSOD1^G93A^ induces rapid morphological and physiological alterations in mitochondria. Because increased action potential firing is one the first pathological events detected in our *in vitro* model system (Fritz et al., 2013; Rojas et al., 2014), we tested whether reducing hyper-excitability with the Na_v_ channel blocker spermidine (10 μM) is capable of preventing the morphological alterations of neuronal mitochondria induced by ACM-hSOD1^G93A^. Interestingly, electron microscopy showed that co-application of ACM-hSOD1^G93A^ and spermidine for 4 h abrogates alterations in the morphology of neuronal mitochondria (**Figures 5A,B**), without changing the number of mitochondria (**Figure 5C**). We also found that chronic exposure of ACM-hSOD1^G93A^ plus spermidine also prevents mitochondrial swelling (Supplementary Figure S7).

### Order of Pathological Events, and Interplay between ROS and Mitochondria

**FIGURE 6:**
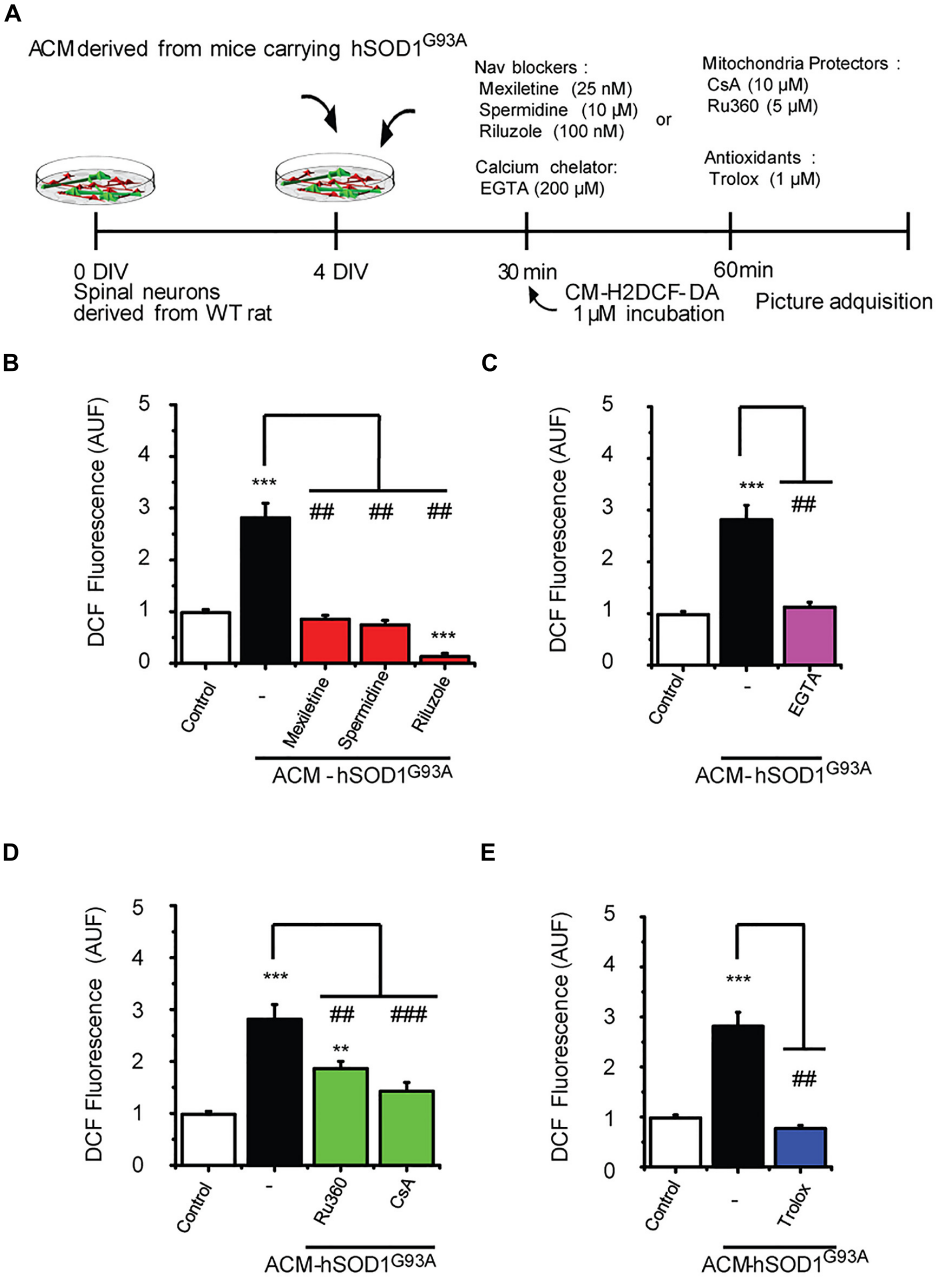
**Na_v_ channel blockers, calcium chelator, mitochondria protectors, and antioxidants prevent DCF fluorescence induced by ACM-hSOD1^G93A^. (A)** Flow diagram of experiment. Spinal cultures (4 DIV) were exposed for 30 min to ACM-hSOD1^G93A^ alone or together with Na_v_ channel blockers: mexiletine (25 nM), spermidine (10 μM), or riluzole (100 nM); calcium extracellular chelator EGTA (200 μM); mitochondria protectors: CsA (10 μM) or Ru360 (5 μM); or the antioxidant Trolox (1 μM). Next, cultures were incubated with the membrane permeable ROS/RNS probe CM-H2DCF-DA and DCF fluorescence was measured 30 min later in neurons using a combination of real-time fluorescence and phase-contrast imaging. **(B–E)** Graphs showing the intensity (arbitrary unit fluorescence; AUF) of DCF fluorescent cells after being treated with ACM-hSOD1^G93A^ alone or with the diverse Na_v_ channel blockers **(B)**, calcium chelator EGTA **(C)**, mitochondria protectors **(D)**, or antioxidant Trolox **(E)**. Values represent mean ± SEM from at least three independent experiments performed in duplicate, analyzed by One-Way ANOVA followed by a Tukey *post hoc* test. ^∗∗^*p* < 0.01, ^∗∗∗^*p* < 0.001 relative to control conditions, and ##*p* < 0.01, ###*p* < 0.001 compared to DCF fluorescence with ALS-causing ACM to at 4 DIV without blockers.

**FIGURE 7:**
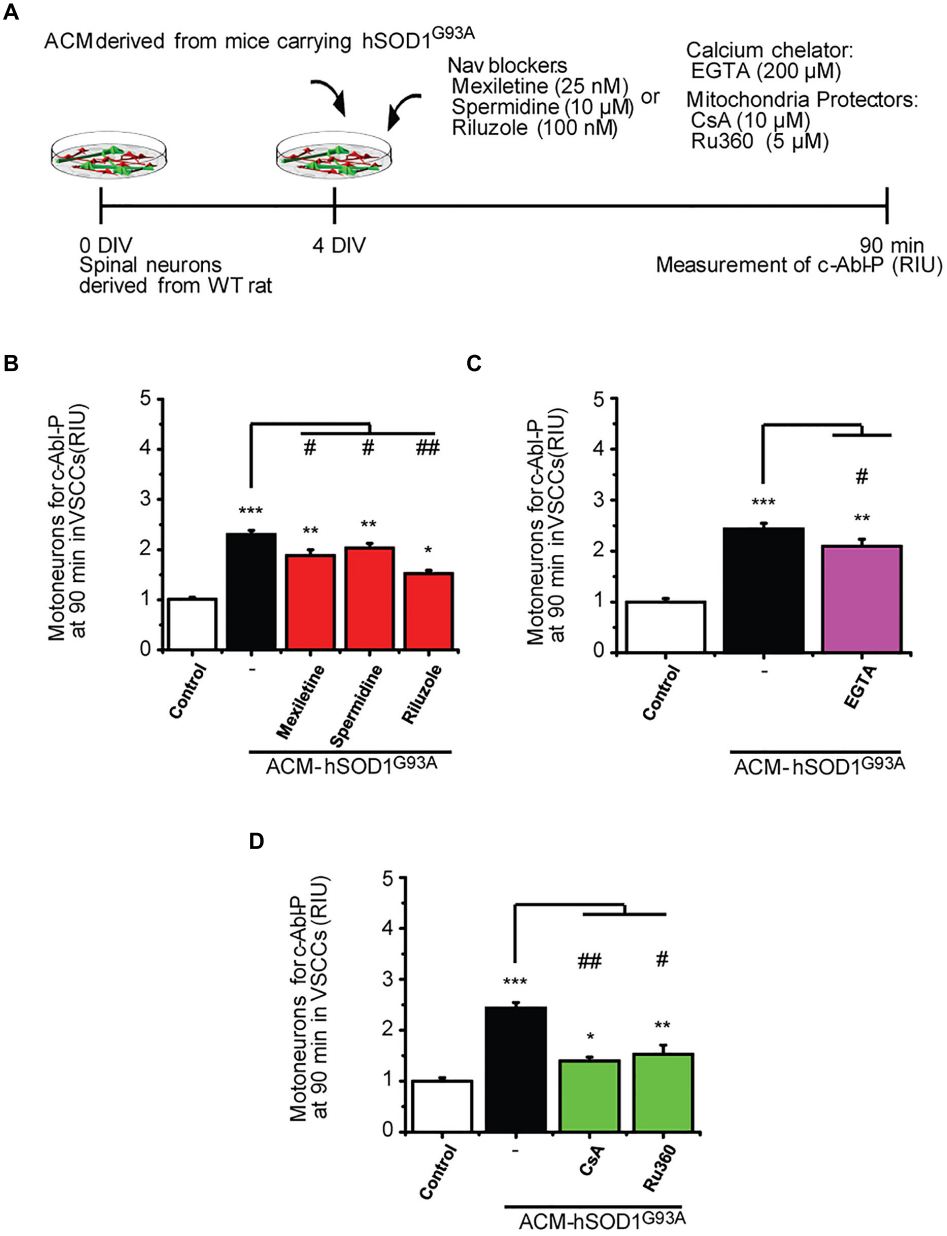
**Na_v_ channel blockers, calcium chelator, mitochondria protectors, and antioxidants reduce c-Abl activation induced by ACM-hSOD1^G93A^. (A)** Flow diagram of experiment. Spinal cultures (4 DIV) were exposed for 90 min to ACM-hSOD1^G93A^ alone or together with Na_v_ channel blockers: mexiletine (25 nM), spermidine (10 μM), or riluzole (100 nM); calcium extracellular chelator EGTA (200 μM); mitochondria protectors: CsA (10 μM) or Ru360 (5 μM). Next, immunostaining was performed to detect phosphorylated c-Abl in motoneurons (MAP2^+^/SMI32^+^ cells). **(B–D)** Graphs showing the c-Abl-P intensity after being treated with ACM-hSOD1^G93A^ alone or with the diverse Na_v_ channel blockers **(B)**, calcium chelator EGTA **(C)**, or mitochondria protectors **(D)**. Values represent mean ± SEM from at least three independent experiments performed in duplicate, analyzed by One-Way ANOVA followed by a Tukey *post hoc* test. ^∗^*p* < 0.05, ^∗∗^*p* < 0.01, ^∗∗∗^*p* < 0.001 relative to control conditions, and #*p* < 0.05, ##*p* < 0.01 compared to DCF fluorescence with ALS-causing ACM at 4 DIV without blockers or antioxidants.

Our data strongly indicate that upon ACM-hSOD1^G93A^ exposure the neuron’s hyper-excitability is the first alteration to occur (at 15–30 min), whereas activation of c-Abl is the last event (at 60–90 min); by contrast, calcium influx, ROS generation and mitochondrial swelling and membrane depolarization are all intermediate events, detectable starting at 30 min after ACM-hSOD1^G93A^ exposure. To determine the exact order of these pathological events and mechanisms whereby c-Abl activation mediates pathogenesis and death of motoneurons, we studied the interplay between mitochondrial structure and membrane physiology, oxidative stress, and c-Abl activation. For this, ROS/RNS production (**Figure 6**) and c-Abl activation (**Figure 7**) were measured in cultures that were exposed to ACM-hSOD1^G93A^ alone or in the presence of multiple pharmacological compounds. We used blockers of Na_v_ channels (25 nM mexiletine, 100 nM riluzole and 10 μM spermidine; as in Fritz et al., 2013; Rojas et al., 2014); chelation of extracellular calcium (200 μM EGTA) to prevent calcium influx; and antioxidants (1 μM Trolox; as in **Figure 3**). To intent to prevent mitochondrial alterations, we used Ru360, a selective inhibitor of calcium uptake in mitochondria (Matlib et al., 1998), as well as cyclosporin A (CsA) to inhibit the mPTP (Crompton et al., 1988, 1998; Broekemeier et al., 1989). For ROS/RNS production, we measured intracellular DCF fluorescence in spinal cord cultures at 60 min of exposure to ACM-hSOD1^G93A^ (**Figure 6A**), at the peak of DCF fluorescence levels as we have shown previously (Rojas et al., 2014). In agreement with our previous work (and repeated here for clarity), we found that co-incubation of ACM-hSOD1^G93A^ with diverse the Na_v_ channel blockers mexiletine, riluzole or spermidine effectively inhibits the increase in DCF fluorescence levels induced by the toxic ACM (**Figure 6B**). In addition, we found that the calcium chelator EGTA resulted in lower intracellular DCF fluorescence levels to degrees similar to that observed in untreated cultures (**Figure 6C**). Co-application of ACM-hSOD1^G93A^ with mitochondrial protectors also strongly reduced DCF fluorescence; cyclosporin A being more effective than Ru360 (**Figure 6D**). These results indicate that ROS/RNS is at least in part released by mitochondria when neurons are exposed to ACM-hSOD1^G93A^. We used several control, including ACM-hSOD1^G93A^ plus Trolox which shows, as expected, that ROS/RNS production was completely prevented (**Figure 6E**). We also found that incubation of spinal cord cultures with ACM-hSOD1^WT^ alone or together with EGTA, cyclosporin A or Ru360 did not affect DCF fluorescence levels (Supplementary Figures S8A–C); the finding that co-incubation of ACM-hSOD1^WT^ with the Na_v_ channel blockers mexiletine, riluzole or spermidine also does not alter DCF fluorescence levels was shown previously (Rojas et al., 2014).

To uncover the upstream events that contribute to c-Abl activation in motoneurons and are mediated by ACM-hSOD1^G93A^, we co-applied ACM from astrocytes expressing hSOD1^G93A^ plus the different compounds (**Figure 7A**); phosphorylation of c-Abl was measured at 90 min (i.e., at the peak of c-Abl phosphorylation as shown in **Figure 1D**_1_). In **Figure 3** we already showed that application of the antioxidants Trolox, esculetin or tiron prevent the increase in phosphorylation of c-Abl. We also tested the effects of Na_v_ channel blockers on ACM-induced c-Abl activation, and find that co-application of ACM-hSOD1^G93A^ plus mexiletine, spermidine or riluzole to spinal cord cultures partly prevents c-Abl phosphorylation in motoneurons (**Figure 7B**). Finally, we find that while the calcium chelator EGTA slightly reduced ROS/RNS production (**Figure 7C**), the mitochondrial protectors Ru360 or cyclosporin A (**Figure 7D**) strongly prevent the c-Abl phosphorylation induced by ACM-hSOD1^G93A^. Incubation of spinal cord cultures with ACM-hSOD1^WT^ alone or together with any of the above indicated compounds did not alter c-Abl phosphorylation levels (Supplementary Figures S8D–H). Collectively, our data indicate that application of ACM-hSOD1^G93A^ leads to the Na_v_-channel-mediated hyper-excitability and calcium influx that trigger mitochondrial swelling and membrane depolarization; the impaired mitochondria contribute, at least in part, to ROS/RNS production that leads to activation of a lethal c-Abl signaling cascade.

## Discussion

### Deciphering the Sequence of Pathological Events in Motoneurons with Use of the ACM-hSOD1^G93A^ Model System

Amyotrophic lateral sclerosis is, at least in part, a non-cell-autonomous disease in which astrocytes that express ALS-causing genes contribute to disease pathogenesis (Clement et al., 2003; Ilieva et al., 2009). Compelling evidence documents that primary astrocytes that express mutant SOD1-expressing and are derived from mice (Nagai et al., 2007; Di Giorgio et al., 2007; Castillo et al., 2013; Fritz et al., 2013; Rojas et al., 2014), rats (Vargas et al., 2006; Cassina et al., 2008), and humans (Marchetto et al., 2008) selectively kill motoneurons, but spare interneurons. Remarkably, astrocytes that are differentiated from neuronal progenitor cells (NPCs) derived either from post-mortem spinal cord tissue or skin biopsies from FALS (SOD1 and C9orf72) and sALS patients, also display non-cell-autonomous toxicity, and selectively kill motoneurons in a co-culture model system (Haidet-Phillips et al., 2011; Meyer et al., 2014; Re et al., 2014). Several pathological phenotypes observed in ALS patients and animal models are recapitulated in these *in vitro* neuron-astrocyte co-cultures, including those that are based on hyper-excitability (Fritz et al., 2013; Rojas et al., 2014), mitochondrial dysfunction (Cassina et al., 2008), and nitroxidative stress (Cassina et al., 2008; Marchetto et al., 2008; Rojas et al., 2014). However, the sequence of pathological events involved in these changes has not been systemically studied. We believe that unraveling the sequence has been difficult because the co-cultures used to study neuron-astrocyte interactions employ neurons that grow on a feeder layer of astrocytes, hereby precluding assessment of the temporal interplay between pathogenic events (Vargas et al., 2006; Di Giorgio et al., 2007; Nagai et al., 2007; Cassina et al., 2008; Marchetto et al., 2008; Haidet-Phillips et al., 2011; Meyer et al., 2014; Re et al., 2014). Nagai et al. (2007) establishes a unique *in vitro* ALS model system, and show that exposure of spinal cultures to conditioned media obtained from astrocytes that express mutated human SOD1 kills primary motoneurons, as well as motoneurons derived from embryonic mouse stem cells. We used (with some adaptions; see Materials and Methods) this *in vitro* model system (Nagai et al., 2007), along with pharmacological interventions and time-dependent measurements at the single-cell level, to systematically unravel the types and temporal order of pathological events induced in motoneurons by ALS-ACM. In addition, the highly diluted ACM-SOD1^G93A^ that we use (see Materials and Methods) enables us to show that motoneuron death is triggered by specific soluble toxic mediator(s), and not by the lack of survival factors. Making use of electrophysiological recordings, calcium imaging and fluorescent probes to detect ROS/RNS, and immunostaining to identify surviving interneurons and motoneurons, we had previously documented that exposure of primary WT spinal cord cultures to ACM-hSOD1^G93A^ leads to a rapid increase in the excitability of neurons (detected already at 15–20 min) and to influx of calcium, which in turn generates intracellular ROS/RNS followed by specific and robust motoneuron death (∼50%) within a matter of days (Fritz et al., 2013; Rojas et al., 2014). In the current study, we have studied the mechanisms that underlie astrocyte-mediated toxicity of motoneurons in more detail, with use of a variety of methods, including ROS/RNS detection with the DCF fluorescent probe; immunostaining to detect c-Abl phosphorylation and the ratio of interneurons:motoneurons; and two different fluorescent probes (TMRM and mitoDsRed2) plus electron microscopy to determine mitochondrial function and structure, respectively. We provide evidence here that toxic factors released by hSOD1^G93A^–expressing astrocytes first increase Na_v_-channel-mediated excitability in neurons, which in turn increases calcium influx, and triggers functional and structural changes in mitochondria. Our data indicate that ROS/RNS, generated at least in part through mitochondrial alterations, activate c-Abl signaling. Although mitochondria are a main source of ROS production for most cells, it has been well established that ROS can be produced at multiple sites in mammalian cells, including by NADPH oxidase (Nox), amino acid oxidases, cytochrome P450 enzymes, cycloxygenases, lipoxygenases and xanthine oxidase (Turrens, 2003). Of interest is also that ROS produced by these different sources does not always activate pathophysiological cellular processes, but can also function as a beneficial signaling messenger (Massaad and Klann, 2011). In ALS pathology, toxic ROS can be produced by dysfunctional mitochondria (Tan et al., 2014; and see below), as well as through Nox enzymes (Wu et al., 2006; Marden et al., 2007; Harraz et al., 2008). Additional studies, which are out of the scope of this present study, should be performed to identify which other source(s), in addition to mitochondria, could be involved in the production of ROS in the ACM-hSOD1^G93A^ model system. Our findings collectively lead us to conclude that the key hallmarks of ALS, including ROS/RNS production and mitochondrial swelling and membrane depolarization, are recapitulated in our *in vitro* neuron-astrocyte model. We predict that our focus on elucidating the mechanisms of non-cell-autonomous motoneuron pathology and death, and on the contribution of Na_v_-channels, ROS/RNS, mitochondria and the c-Abl pathway, will yield advances for the use and/or development of therapeutic interventions for this devastating disease.

### Dysfuntional Mitochndria as a Source of ROS/RNS

Mitochondria participate in energy metabolism, intracellular calcium homeostasis, production of intracellular ROS, and regulation of apoptosis (Nicholls, 2009; Rasola and Bernardi, 2011; Maryanovich and Gross, 2013). Morphological and functional abnormalities in mitochondria are a common feature of ALS, and are detected in biopsied and post-mortem tissue from symptomatic sALS and fALS patients, as well as in mutant SOD1 mouse models and the cell cultures derived from these mice (reviewed in Manfredi and Xu, 2005; Bento-Abreu et al., 2010; Grosskreutz et al., 2010; Tan et al., 2014). Degenerated, vacuolized and swollen mitochondria are detected in transgenic mice that express mutations in SOD1, around the time when clinical symptoms appear (Dal Canto and Gurney, 1995; Wong et al., 1995; Kong and Xu, 1998). Moreover, ultra-structural analysis reveals that mitochondrial swelling is detected in spinal cord motoneurons of hSOD1^G93A^ transgenic mice at P14, 2–3 months *prior to* the time that motoneurons degeneration is visible, and before clinical symptoms are manifest (Bendotti et al., 2001); these findings underscore early mitochondrial abnormalities in this mouse model and suggest that mitochondrial dysfunction might contribute to motoneuron pathology. Electron microscopy also shows that in our *in vitro* ALS model system mitochondrial swelling is an early event in motoneuron pathogenesis, and is induced by soluble mediator(s) secreted by hSOD1^G93A^-expressing astrocytes. Swelling of mitochondria may be caused by activation of mPTP, a voltage-dependent, high conductance mega channel whose opening is critically regulated and triggered by various processes, including matrix calcium accumulation, ROS, and adenine nucleotide depletion (Miller, 1998; Kroemer and Reed, 2000; Rasola and Bernardi, 2011; Bernardi and Di Lisa, 2015).

Our findings with cyclosporine A and Ru360, along with results from use of mitochondrial fluorescent probes, suggest that hSOD1^G93A^ astrocyte-triggered abnormalities are caused, at least in part, by the accumulation of calcium in mitochondria; the calcium uptake could induce the opening of mPTP in motoneurons which is followed by a rapid loss of ΔΨm, uncontrolled matrix calcium and oxidative species release, matrix swelling and eventually rupture of the outer membrane, and release of apoptogenic factors (Rasola and Bernardi, 2011). Thus the opening of mPTP may induce both apoptotic and necrotic death signal

Because mitochondria predominantly generate superoxide anion (O_2_^∙-^) as a by-product of the respiratory chain functioning (Murphy, 2009), which is not detectable by the DCF probe used in this study (Myhre et al., 2003), our findings indicate that other reactive oxygen and nitrogen species contribute to the motoneuron pathology observed in our model system. On the other hand, DCF does detect hydrogen peroxide (H_2_O_2_; in combination with cellular peroxidases), hydroxyl radicals (^∙^OH), and peroxynitrite (ONOO**^-^**; Estévez et al., 1999; Myhre et al., 2003; Gomes et al., 2005; Martin et al., 2007; Kalyanaraman et al., 2012). Of particular interest is ONOO**^-^** which is formed from ∙NO and O_2_^∙-^ and is strongly implicated in several models of ALS, but is predominantly detected by the amounts of 3-nitrotyrosine formation in proteins (Abe et al., 1997; Bruijn et al., 1997; Beckman et al., 2001; Radi, 2004; Wu et al., 2006). Reactive and SOD1^G93A^-expressing astrocytes are a major source of ∙NO, which then can target neighboring cells and affects the survival of WT motoneurons (Vargas et al., 2006; Cassina et al., 2008). Based on these reports, we hypothesize that conditioned media derived from hSOD1^G93A^-expressing astrocytes includes ∙NO and/or leads to the generation of ∙NO in the WT astrocytes that are present within spinal cord cultures upon acute exposure of the toxic ACM; this ∙NO in turn permeates into neurons, where it interacts with the intracellular O_2_^∙-^ produced by mitochondria, and forms ONOO**^-^**.

### Activation of c-ABL in ALS

Active c-Abl is implicated in a variety of neurodegenerative diseases, including Alzheimer’s (Alvarez et al., 2004; Cancino et al., 2008; Estrada et al., 2011; Gonzalez-Zuñiga et al., 2014) and Parkinson’s diseases (Imam et al., 2011). Recently, Katsumata et al. (2012) reported a significant increase in c-Abl expression in post-mortem spinal cord tissues from sALS patients, and in the spinal cord of symptomatic hSOD1^G93A^ mice; they also demonstrated that oral administration of the c-Abl inhibitor dasatinib to animals aged P56–P120 attenuates motoneuron loss, alleviates motor dysfunction, and significantly increases lifespan (Katsumata et al., 2012). In the current study, we also document that phosphorylation of c-Abl is significantly increased in the brain and spinal cord of symptomatic hSOD1^G93A^ mice; furthermore, we show that activated c-Abl is detectable in symptomatic hSOD1^G86R^ mice. Importantly, exposure of WT spinal cord cultures to conditioned media derived from astrocytes expressing SOD1 (SOD1^G93A^ or SOD1^G86R^) TDP43 (TDP43^A315T^) mutants, activates the c-Abl tyrosine kinase in WT motoneurons. Our results show that activation of c-Abl in two completely unrelated ALS models leads to the death of motoneurons via non-cell autonomous processes. We used our *in vitro* ACM-hSOD1^G93A^ model system to investigate the potential causes of c-Abl activation in ALS: in agreement with earlier studies, which report that oxidative stress can induce c-Abl activation (van Etten, 1999; Klein et al., 2011; Schlatterer et al., 2011a), we found that ROS (H_2_O_2_) increases c-Abl phosphorylation, and that co-application of antioxidants plus ACM-hSOD1^G93A^ strongly reduce activation of this kinase, in a manner similar to that of the c-Abl inhibitor STI571. Moreover, we also show that reducing c-Abl activation by antioxidants as wells as by STI571 protects astrocyte-mediated toxicity in motoneurons. Experiments with use of mitochondrial protectors (cyclosporine A and Ru360) point to a key role for mitochondrial-produced ROS in the activation of c-Abl. Based on these findings, we hypothesize that inhibition of ROS production (by protecting mitochondrial dysfunction or by application of antioxidants) in ALS animals might largely prevents motoneuron pathology and significantly extends the lifespan of these mice.

### Hyper-Excitability is a Primary Pathological Event in Astrocyte-Triggered Motoneuron Degeneration

Our current and earlier findings with use of the ACM *in vitro* model (Fritz et al., 2013; Rojas et al., 2014) strongly implicate hyper-excitability as a critical pathological event in astrocyte-triggered motoneuron degeneration, and show that it occurs upstream of mitochondrial impairment, ROS production, and c-Abl activation. The use of electrophysiological recordings in motoneurons in acute slice preparations of neonatal SOD1^G93A^ mice (P4–P10), reveals that increased excitability (van Zundert et al., 2008) precedes the mitochondrial damage that is detected at 2 weeks of postnatal life (Bendotti et al., 2001). Additional studies also show that electrophysiological abnormalities in motoneurons are the earliest physiological alterations detected in diverse ALS rodent models (Kuo et al., 2005; Bories et al., 2007; Pambo-Pambo et al., 2009; Quinlan et al., 2011; van Zundert et al., 2012). Hyper-excitability has been reported in fALS and sALS patients (van Zundert et al., 2012), and also in induced pluripotent stem cell-derived motoneurons (i-motoneurons) generated from ALS patients that harbor mutations in SOD1, C9orf72, and FUS/TLS (Wainger et al., 2014). Generation of action potentials, and hence of membrane excitability, is regulated by the number and functioning of Na_v_ and K_v_ channels on neuronal cells. Currently, we have little insight into the mechanism responsible for hyper-excitability, but we do know that it is related to an increase in persistent Na_v_ channel currents (van Zundert et al., 2008, 2012; Fritz et al., 2013; Pieri et al., 2013), and to a reduction in the amplitude of the delayed-rectifier K channel current (Wainger et al., 2014). The possibility that a factor(s) released by ALS astrocytes regulates the function and/or expression of these ion channels is intriguing, and its identification is of great importance. Independent of the mechanisms, pharmacological assays *in vitro* show that decreasing the activity of Na_v_ channels of motoneurons by co-application of ACM-hSOD1^G93A^ plus the Na_v_ channel blockers mexiletine, riluzole or spermidine (Fritz et al., 2013; Rojas et al., 2014; current study), or by increasing K_v_ channel activity via treatment of ALS i-motoneurons with retigabine (Wainger et al., 2014), inhibits hyper-excitability and improves motoneuron survival. These compounds may also be promising candidate drugs for attenuating pathogenesis and delaying the onset of disease-specific symptoms of fALS and sALS patients; neurophysiological studies in both types of patients have uncovered neuronal hyper-excitability (van Zundert et al., 2012). In addition, because these patients are similar to each other in terms of other pathological events (e.g., mitochondrial impairment and production of ROS), clinical symptoms, and the benefits gained from treatment with riluzole (a non-specific Na_v_ channel blocker; Bellingham, 2011), we hypothesize that motoneuron hyper-excitability represents a general feature of sALS and fALS patients, and that motoneuron death is triggered by activation of a common fatal pathogenic signaling pathway (van Zundert et al., 2012). Further support for this hypothesis will come from results of phase II trial that have been initiated by the ALS Therapy Alliance (ATA) and the Northeast ALS (NEALS) Consortium with use of mexiletine to treat sALS patients. Our findings also underscore the need for further tests in diverse ALS animal models and in ALS patients with the compounds that successfully prevent motoneuron damage in ALS neuron-astrocyte *in vitro* systems: within this context, the anti-oxidants Trolox and esculetin are especially promising because of their strong antioxidant activity and their ability to cross the blood-brain-barrier (Barber and Shaw, 2010).

## Author Contributions

FR and SA performed MN survival experiments. NC executed DCF measurement. DG, FR, EA, AM. performed c-Abl immunostainings *in vitro* and *in vivo*. EF and FC performed EM studies. NC, DH, EA executed TMRM and DsRed2 assays. DG and AM performed western blot assays. All analyzed the data and wrote the manuscript.

## Conflict of Interest Statement

The authors declare that the research was conducted in the absence of any commercial or financial relationships that could be construed as a potential conflict of interest.

## Supplementary material

The Supplementary Material for this article can be found online at: http://journal.frontiersin.org/article/10.3389/fncel. 2015.00203/abstract
