# Supplementary material for: Reactive oxygen species trigger motoneuron death in non-cell-autonomous models of ALS through activation of c-Abl signaling
Source: Front Cell Neurosci. 2015 Jun 9;9:203. doi: 10.3389/fncel.2015.00203 (PMC4460879; doi:10.3389/fncel.2015.00203)
Supplement: Supplementary file 1 [file Data_Sheet_1.PDF]

SUPPLEMENTARY FIGURE LEGENDS

SUPPL FIG 1 | Phosphorylated c-Abl is increased in the spinal cord and brain of symptomatic SOD1^{G93A} mice.

(**A**) Tissue for western blot assays and immunostaining was used from wild-type and symptomatic hSOD1^{G93A} mice (>P120) to detect phosphorylated c-Abl (phospho-Tyr412). (**B$_{1-2}$**) Whole lysates from spinal cords (**B$_1$**) and brains (**B$_2$**) derived from wild type and SOD1^{G93A} mice were analyzed by western blots, using antibodies against c-Abl and phosphorylated c-Abl. N-Cadherin was used as loading control. (**B$_{3-4}$**) Quantification of the western blots from spinal cords (**B$_3$**) and brains (**B$_4$**) derived from wild type and SOD1^{G93A} mice. (**C**) Immunofluorescence of ventral sections from lumbar spinal cord for either wild type or SOD1^{G93A} mice.

SUPPL FIG 2 | Phosphorylated c-Abl is augmented in spinal cord and motor cortex of symptomatic SOD1^{G86R} mice.

(**A**) Tissue for western blot assays and immunostaining was used from wild-type and symptomatic SOD1^{G86R} mice (P95) to detect phosphorylated c-Abl (phospho-Tyr412). (**B$_{1-2}$**) Whole lysates from spinal cords (**B$_1$**) and motor cortex (**B$_2$**) derived from wild type and SOD1^{G86R} mice were analyzed by western blots, using antibodies against c-Abl and phosphorylated c-Abl. N-Cadherin was used as loading control. (**B$_{3-4}$**) Quantification of the western blots from spinal cords (**B$_3$**) and motor cortex (**B$_4$**) derived from wild type and SOD1^{G86R} mice. Values represent mean ± SEM from at least 3 independent experiments performed in duplicate, analyzed by t-test: ***p<0.001 vs. control. Values represent mean ± SEM from at least 3 independent experiments performed in duplicate, analyzed by t-test: ***p<0.001 vs. control. (**C**) Immunofluorescence of ventral sections from lumbar spinal cord for either wild type or SOD1^{G86R} mice.

SUPPL FIG 3 | Exposure of primary spinal cord cultures to ACM derived from $SOD1^{G93A}$, $SOD1^{G93A}$ or $TDP43^{A315T}$ increases lethal c-Abl phosphorylation in motoneurons.

(**A**) Flow diagram of experiment. Primary wild-type (WT) rat spinal cord cultures (4 DIV) were exposed to ACM derived from transgenic mice overexpressing $SOD1^{G93A}$ (ACM-$SOD1^{G93A}$), $SOD1^{G86R}$ (ACM-$SOD1^{G86R}$), or $TDP43^{A315T}$ (ACM-$TDP43^{A315T}$) for 0 to 120 minutes. Next cultures were washed, fixed and immunostained with an antibody recognizing phosphorylated c-Abl (Tyr-412) and the SMI-32 antibody to identify motoneurons. (**B**) Graphs showing the c-Abl-P fluorescent intensity (relative unity intensity; RUI) at 0, 30, 60, 90 and 120 min after application of ACM-$SOD1^{G93A}$ (**B_1**), ACM-$SOD1^{G86R}$ (**B_2**), or ACM-$TDP43^{A315T}$ (**B_3**) (red line). Results obtained ACM-$hSOD1^{WT}$ are also included (grey lines). In all experiment, H_2O_2 (200 µM for 20 min) served as positive control (green dot). (**C**) Flow diagram of experiment. Primary wild-type (WT) rat spinal cord cultures (4 DIV) were exposed to ACM-$SOD1^{G93A}$, ACM-$SOD1^{G86R}$, or ACM-$TDP43^{A315T}$. At 90 min (ACM-$SOD1^{G93A}$ and ACM-$SOD1^{G86R}$) or 120 min (ACM-$TDP43^{A315T}$) cultures were washed, fixed and immunostained with an antibody recognizing phosphorylated c-Abl (Tyr-412) and the SMI-32 antibody to identify motoneurons. Motoneuron cell survival ($MAP2^{+}$/$SMI\text{-}32^{+}$) was also measured at 7 DIV (**D**) Graphs showing the c-Abl-P fluorescent intensity (RUI) at 90 min/120 min after application of ACM-$SOD1^{G93A}$ (**D_1**), ACM-$SOD1^{G86R}$ (**D_2**), or ACM-$TDP43^{A315T}$ (**D_3**). (**E**) Graph showing the relative percentage of motoneurons that survived at 7 DIV, after being treated with STI571 and ACM-$SOD1^{G93A}$ (**E_1**), ACM-$SOD1^{G86R}$ (**E_2**), or ACM-$TDP43^{A315T}$ (**E_3**). Values represent mean ± SEM from at least 3 independent experiments performed in duplicate, analyzed by One-Way ANOVA followed by a Tukey *post-doc* test $*p<0.05$, $**p<0.01$,

***$p<0.001$ vs. control: ##$p<0.01$ and ###$p<0.001$ compared to survival with the ALS-causing ACM without STI571.

SUPPL FIG 4 | c-Abl kinase inhibitor STI571 prevents c-Abl phosphorylation in motoneurons, interneurons and glial cells induced by ACM-SOD1^{G93A}.

(**A**) Flow diagram of experiment. ACM-hSOD1^{G93A} was applied to 4 DIV spinal cord cultures acutely (for 90 minutes when c-Abl-P peaks; see Figure 1) or chronically (3 days) either alone or in the presence of c-Abl kinase inhibitor STI571 (1 μM). Percentage of cells positive for c-Abl phosphorylation was measured at 4 and 7 DIV. (**B**) Graphs showing percentage of cells positive for c-Abl-P at 4 DIV when treated acutely (90 min) with ACM-hSOD1^{G93A} alone or ACM-hSOD1^{G93A} plus STI571; motoneurons (**B$_1$**), interneurons (**B$_2$**) and astrocytes (**B$_3$**) were identified by immunostaining (as in Figure 1). (**C**) Same as in B, but c-Abl-P is measured at 7 DIV when treated chronically (3 days) with ACM-hSOD1^{G93A} alone or with STI571. Values represent mean ± SEM from at least 3 independent experiments performed in duplicate, analyzed by One-Way ANOVA followed by a Tukey *post-doc* test. **$p<0.01$, ***$p<0.001$ relative to control conditions. #$p<0.05$, compared to survival with the ALS-causing ACM without STI-571.

SUPPL FIG 5 | Diverse antioxidants effectively reduce DCF fluorescence induced by H_2O_2.

(**A**) Flow diagram of experiment. Spinal cultures (4 DIV) were incubated with the membrane permeable ROS/RNS probe CM-H2DCF-DA and exposed for 20 min to 200 μM H_2O_2 alone or together with the antioxidant Trolox (1 μM), esculetin (25 μM), or tiron (25 μM). DCF fluorescence was measured 20 min later in neurons using a combination of real-time fluorescence and phase-contrast imaging. (**B**) Graphs showing DCF intensity normalized to DCF fluorescence obtained in H_2O_2 treated neurons. Values represent mean ± SEM from at least 2 independent experiments performed in duplicate, analyzed by One-Way ANOVA followed by a Tukey *post-doc* test. #$p<0.05$, and ##$p<0.01$, compared to DCF fluorescence measured in H_2O_2-treated neurons.

SUPPL FIG 6 | Antioxidants reduce c-Abl phosphorylation in motoneurons, interneurons and glial cells induced by ACM-SOD1^{G93A}.

(**A**) Flow diagram of experiment. ACM-SOD1^{G93A} was applied to 4 DIV spinal cord cultures acutely (for 90 minutes when c-Abl-P peaks; see Figure 1) or chronically (3 days) either alone or in the presence of antioxidants Trolox (1 μM), esculetin (25 μM), or tiron (25 μM). Percentage of cells positive for c-Abl phosphorylation was measured at 4 DIV (90 min) or 7 DIV. (**B**) Graphs showing percentage of cells positive for c-Abl-P at 4 DIV when treated acutely (90 min) with ACM-SOD1^{G93A} alone or ACM-SOD1^{G93A} plus the antioxidants; motoneurons (**B$_1$**), interneurons (**B$_2$**) and glial cells (**B$_3$**) were identified by immunostaining (as in Figure 1). (**C**) Same as in B, but c-Abl-P is measured at 7 DIV when treated chronically (3 days) with ACM-SOD1^{G93A} alone or with antioxidants. Values represent mean ± SEM from at least 3 independent experiments performed in duplicate, analyzed by One-Way ANOVA followed by a Tukey *post-doc* test. **p<0.01, ***p<0.001 relative to control conditions. #p<0.05, compared to survival with the ALS-causing ACM without treatment.

SUPPL FIG 7 | Spermidine prevents swelling of neuronal mitochondria when exposed to ACM-hSOD^{G93A}.

(**A**) Representative electron microscopy images of spinal cord cultures treated for 4, 8, 24 and 48 hrs with ACM-hSOD1^{WT}, ACM-hSOD1^{G93A} alone or ACM-hSOD1^{G93A} plus spermidine (1 μM). Scale bar, 0.1 μm. (**B**) Graph showing mitochondrial circularity of cells after treatment with the different ACMs with or without spermidine. (**C**) Graph showing percentage of total mitochondria size area of cultures under the same treatment shown in B, classified in 4 groups: "small" (S; <0.2 μm^2) (**C$_1$**), "medium" (M; 0.21-0.4 μm^2) (**C$_2$**), "large" (L; 0.41-0.6 μm^2) (**C$_3$**), and "extra large" (XL; >0.6μm^2) (**C$_4$**). Values represent mean ± S.E.M. from over 100 mitochondria per condition, analyzed by one-way ANOVA followed by a Tukey post-hoc test. *P< 0.05 relative to ACM-hSOD1^{WT}. ##p<0.01 and ###p<0.001 relative to ACM-hSOD^{G93A}.

SUPPL FIG 8 | ACM-SOD1^{G93A} alone or in the presence of Na$_v$ channel blockers, calcium chelator, mitochondria protectors, or antioxidants do not alter DCF fluorescence or c-Abl-P.

(A) Flow diagram of experiment. Spinal cultures (4 DIV) were exposed for 30 min to ACM-hSOD1^{WT} alone or together with calcium extracellular chelator EGTA (200 μM) or mitochondria protectors: CsA (10 μM) or Ru360 (5 μM) Next, cultures were incubated with the membrane permeable ROS/RNS probe CM-H2DCF-DA and DCF fluorescence was measured 30 min later in neurons using a combination of real-time fluorescence and phase-contrast imaging. (**B-C**) Graphs showing the intensity (arbitrary unit fluorescence; AUF) of DCF fluorescent cells after being treated with ACM-hSOD1^{G93A} alone or with the diverse mitochondria protectors (**B**), or calcium chelator EGTA (**C**). (**D**) Flow diagram of experiment. Spinal cultures (4 DIV) were exposed for

90 min to ACM-hSOD1^{WT} alone or together with Na_v channel blockers: mexiletine (25 nM), spermidine (10 μM), or riluzole (100 nM); calcium extracellular chelator EGTA (200 μM); the antioxidants: Trolox (1 μM), Esculetin (25 μM) or Tiron (25 μM); or mitochondria protectors: CsA (10 μM) or Ru360 (5 μM). (**E-H**) Graphs showing the c-Abl-P intensity after being treated with ACM-hSOD1^{G93A} alone or with the diverse antioxidants (**E**), Na_v channel blockers (**F**), mitochondria protectors (**G**), or calcium chelator EGTA (**H**). Values represent mean ± SEM from at least 3 independent experiments performed in duplicate, analyzed by One-Way ANOVA followed by a Tukey *post-doc* test. All data are non-significant different.

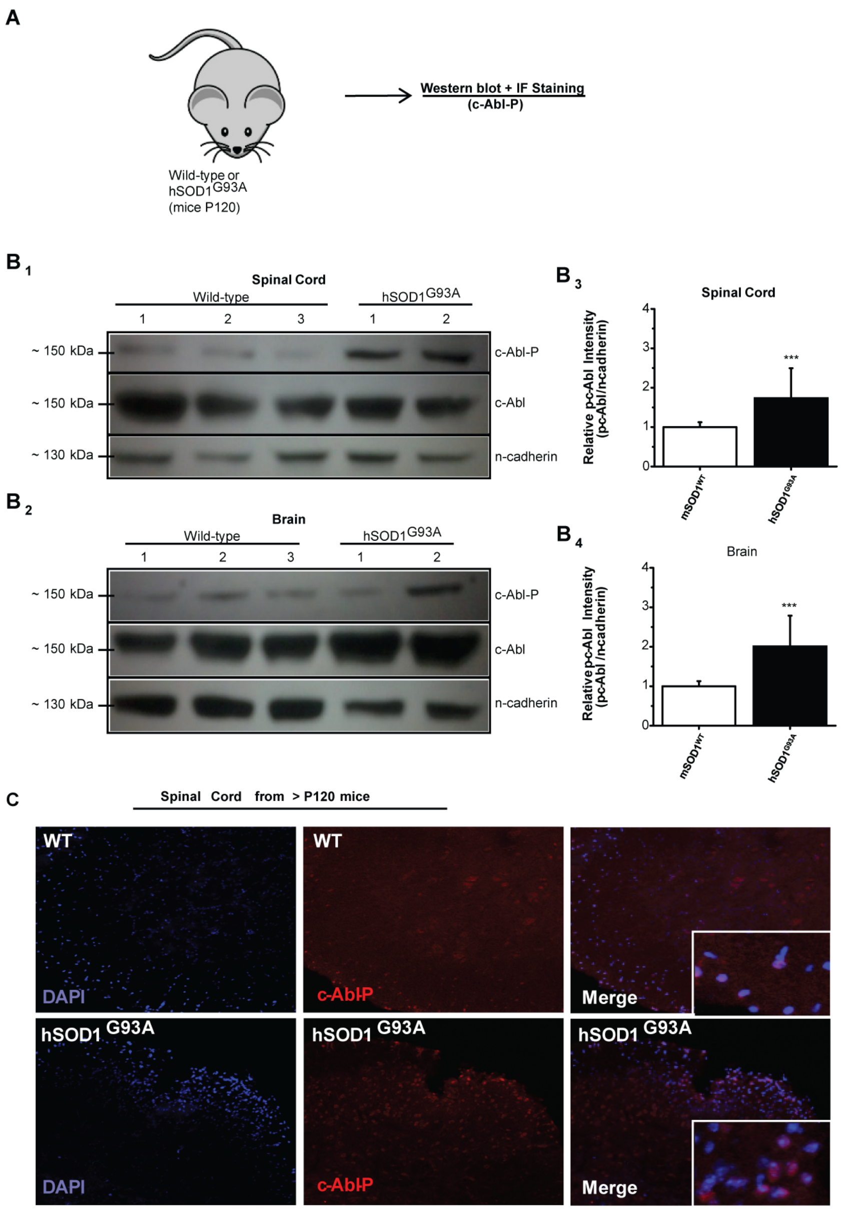

Supplementary Figure 1
Rojas et al., 2015

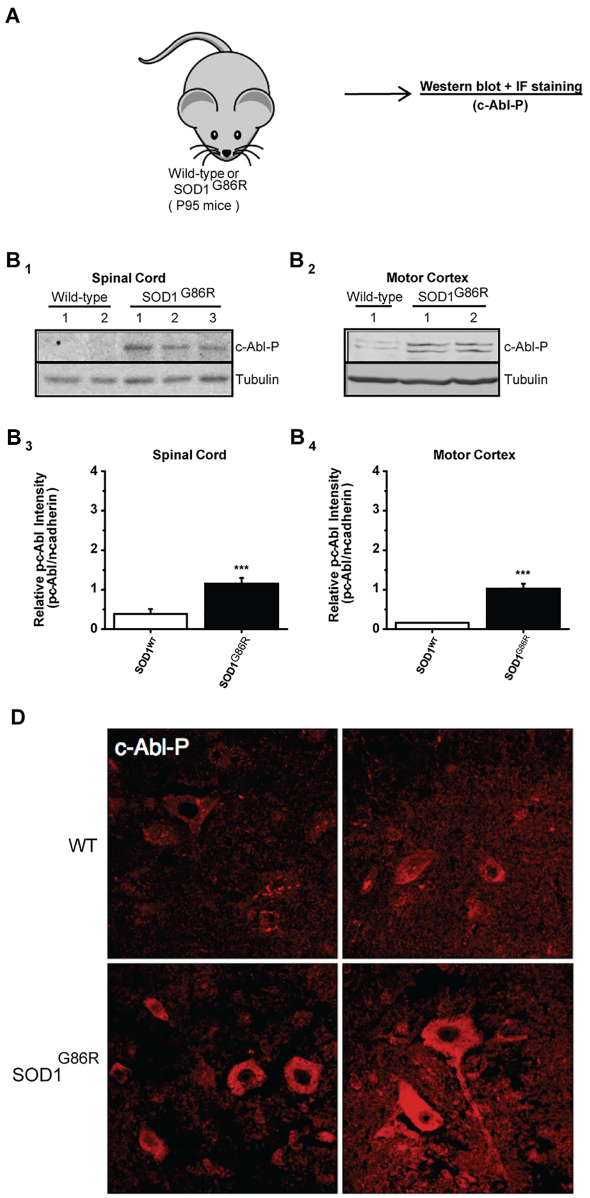

Supplementary Figure 2
Rojas et al., 2015

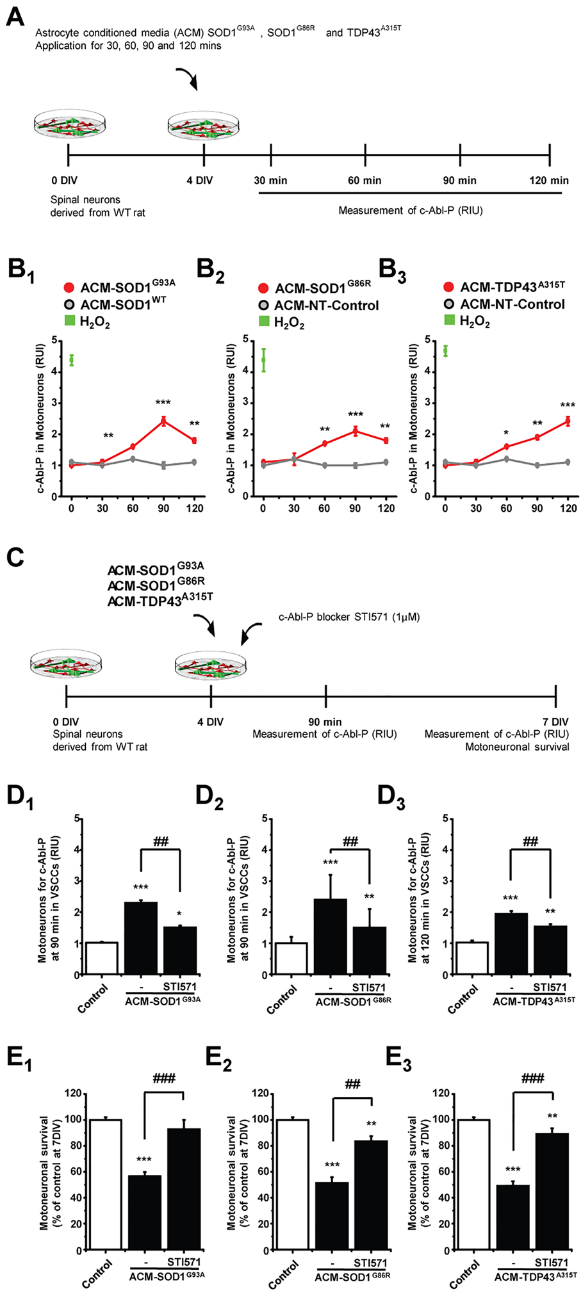

Supplementary Figure 3
Rojas et al., 2015

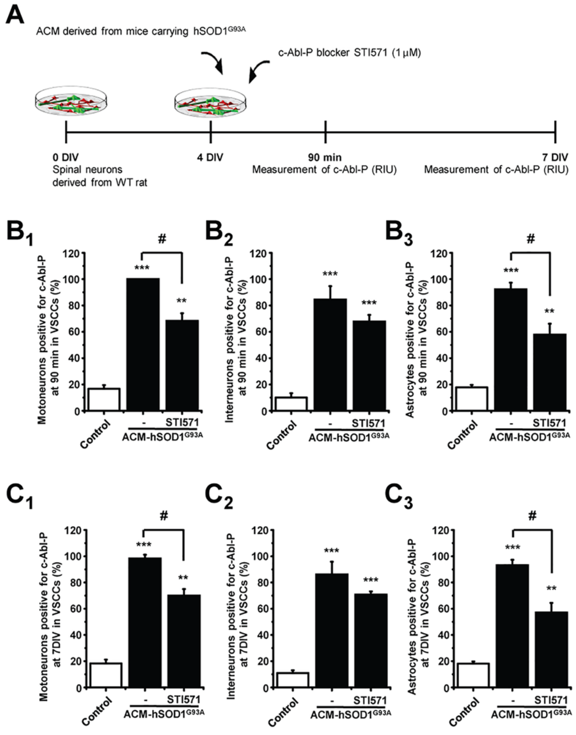

Supplementary Figure 4
Rojas et al., 2015

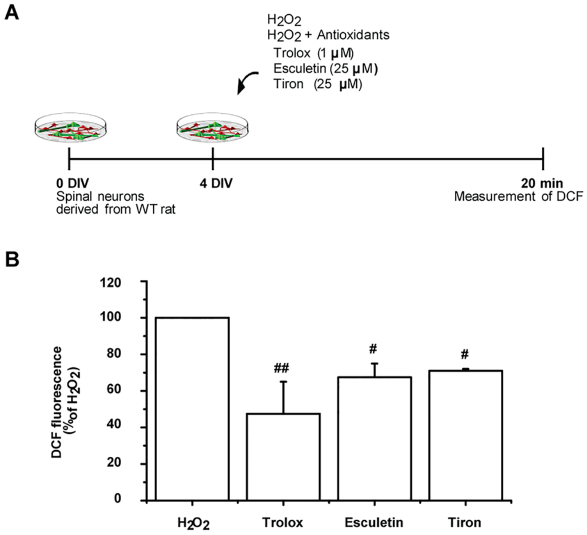

Supplementary Figure 5
Rojas et al., 2015

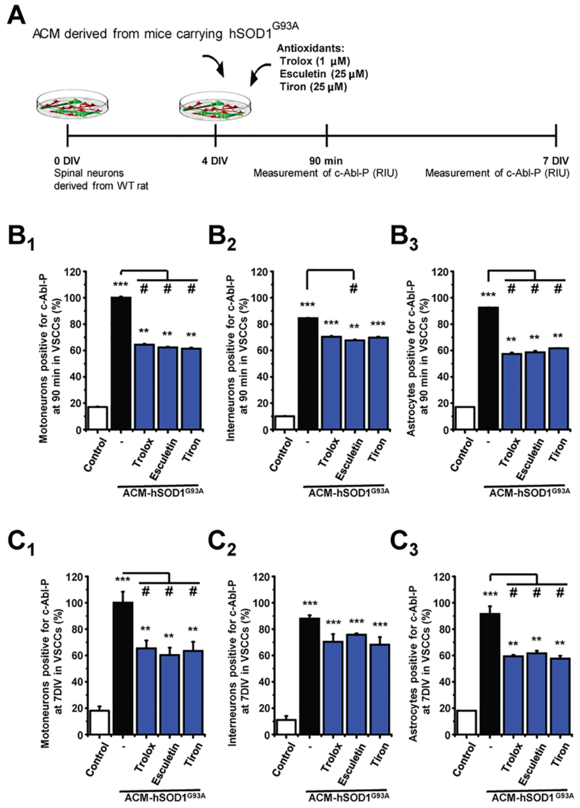

Supplementary Figure 6
Rojas et al., 2015

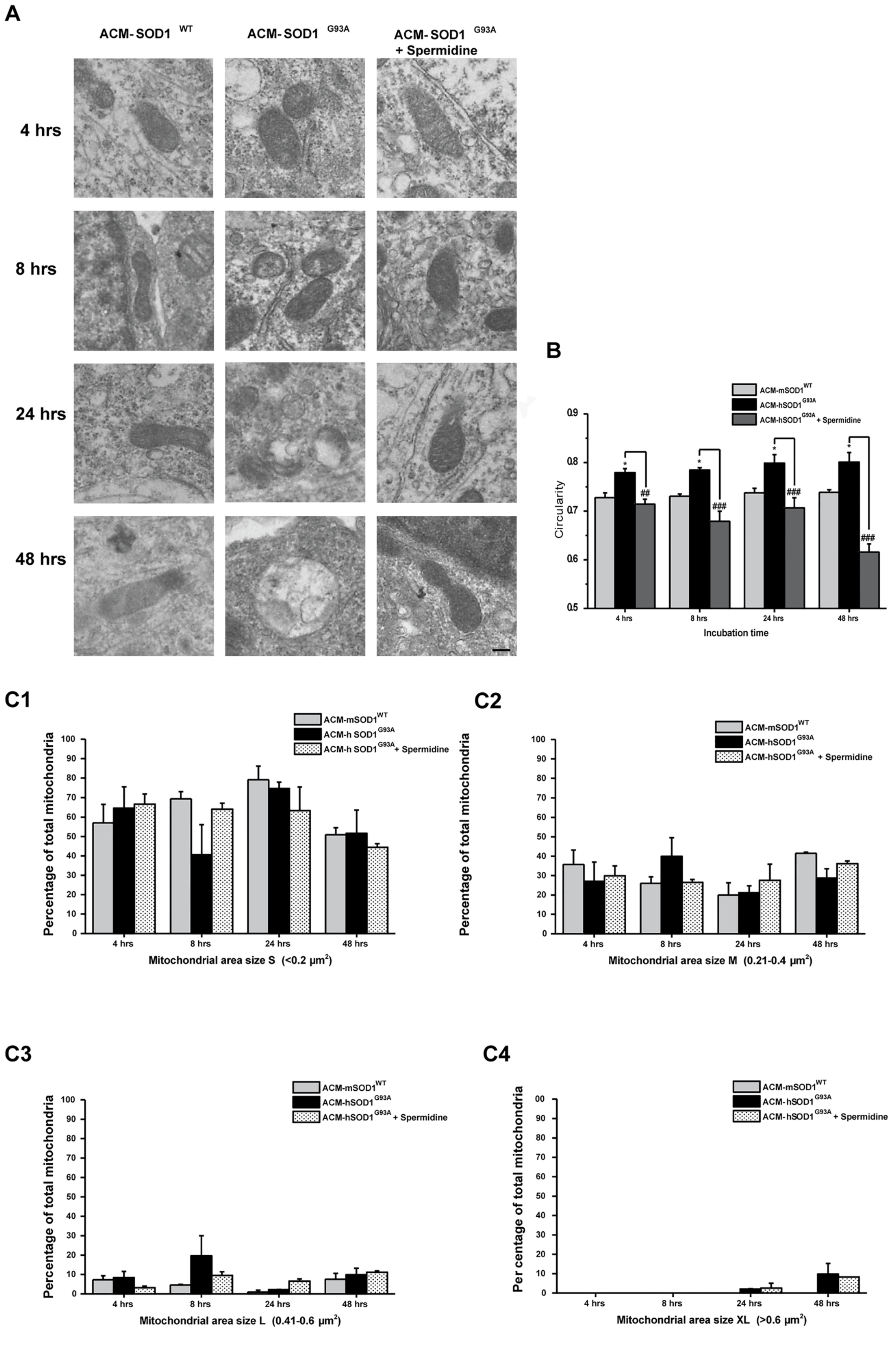

Supplementary Figure 7
Rojas et al., 2015

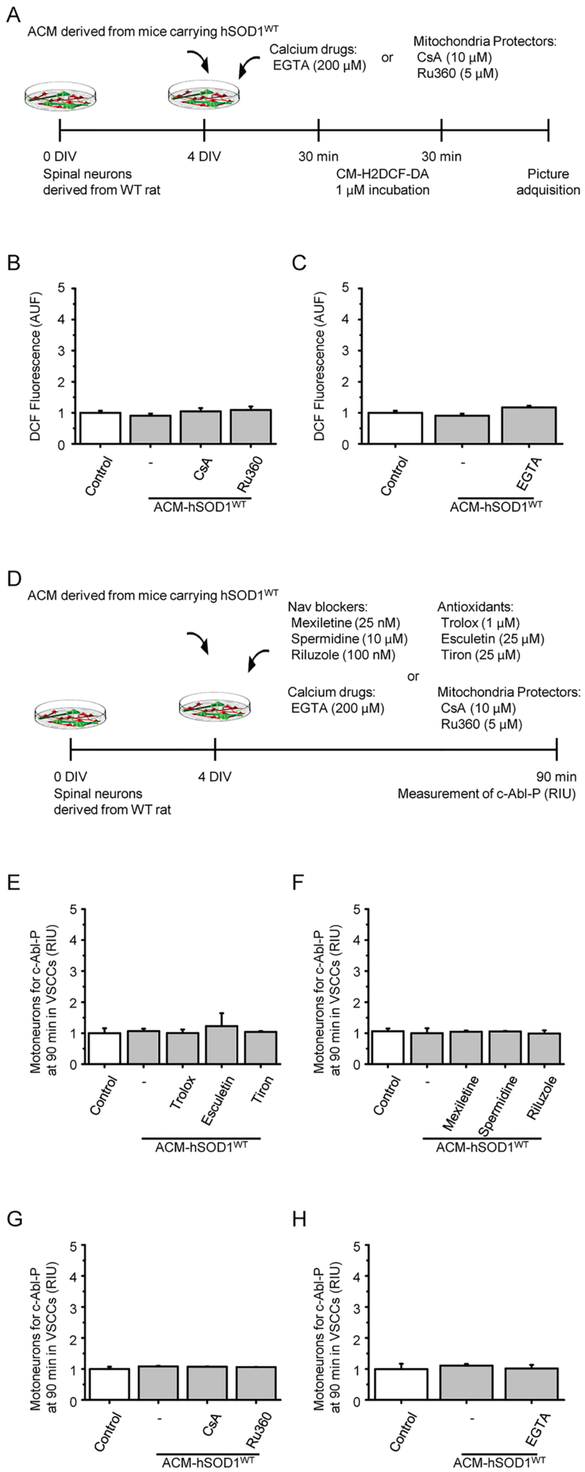

Supplementary Figure 8
Rojas et al., 2015
